# Evolutionary Insights into the Length Variation of DNA Damage Response Proteins Across Eukaryotes

**DOI:** 10.1093/gbe/evaf089

**Published:** 2025-05-19

**Authors:** Dominic Wiredu-Boakye, Laurence Higgins, Ondřej Gahura, Anzhelika Butenko, Guy Leonard, Mark A Freeman, Árni Kristmundsson, Karen Moore, Jamie W Harrison, Shani Mac Donald, Vyacheslav Yurchenko, Bryony A P Williams, Richard Chahwan

**Affiliations:** Faculty of Health and Life Sciences, Clinical and Biomedical Sciences, University of Exeter, Exeter, Devon, UK; Faculty of Health and Life Sciences, Clinical and Biomedical Sciences, University of Exeter, Exeter, Devon, UK; Institute of Parasitology, Biology Centre, Czech Academy of Sciences, České Budejovice, Czechia; Institute of Parasitology, Biology Centre, Czech Academy of Sciences, České Budejovice, Czechia; Life Science Research Centre, Faculty of Science, University of Ostrava, Ostrava, Czechia; Faculty of Sciences, University of South Bohemia, České Budějovice 370 05, Czechia; Department of Biology, University of Oxford, Oxford, UK; Conservation Medicine and Ecosystem Health, Biomedical Sciences, Ross University School of Veterinary Medicine, Basseterre, St. Kitts; Fish Disease Laboratory, Institute for Experimental Pathology, University of Iceland, Reykjavík, Iceland; Faculty of Health and Life Sciences, Clinical and Biomedical Sciences, University of Exeter, Exeter, Devon, UK; Faculty of Health and Life Sciences, Clinical and Biomedical Sciences, University of Exeter, Exeter, Devon, UK; Faculty of Health and Life Sciences, Clinical and Biomedical Sciences, University of Exeter, Exeter, Devon, UK; Life Science Research Centre, Faculty of Science, University of Ostrava, Ostrava, Czechia; Faculty of Health and Life Sciences, Clinical and Biomedical Sciences, University of Exeter, Exeter, Devon, UK; Cancer Immunobiology Laboratory, Institute of Experimental Immunology, University of Zurich, Zurich, Switzerland

**Keywords:** DNA lesions, genome compaction, DNA damage signalling, protein length, intracellular parasites

## Abstract

Across the tree of life, DNA damage response (DDR) proteins play a pivotal, yet dichotomous role in organismal development and evolution. Here, we present a comprehensive analysis of 432 DDR proteins encoded by 68 genomes, including that of *Nucleospora cyclopteri*, an intranuclear microsporidia sequenced in this study. We compared the DDR proteins encoded by these genomes to those of humans to uncover the DNA repair-ome across phylogenetically distant eukaryotes. We also performed further analyses to understand if organismal complexity and lifestyle play a role in the evolution of DDR protein length and conserved domain architecture. We observed that the genomes of extreme parasites such as *Paramicrocytos*, *Giardia*, *Spironucleus,* and certain microsporidian lineages encode the smallest eukaryotic repertoire of DDR proteins and that pathways involved in modulation of nucleotide pools and nucleotide excision repair are the most preserved DDR pathways in the eukaryotic genomes analysed here. We found that DDR and DNA repair proteins are consistently longer than housekeeping and metabolic proteins. This is likely due to the higher number of physical protein–protein interactions which DDR proteins are involved. We find that although DNA repair proteins are generally longer than housekeeping proteins, their functional domains occupy a relatively smaller footprint. Notably, this pattern holds true across diverse organisms and shows no dependence on either lifestyle or mitochondrial status. Finally, we observed that unicellular organisms harbour proteins that are tenfold longer than their human homologues, with the extra amino acids forming interdomain regions with a clearly novel albeit undetermined function.

**Significance**

The recent explosion of genomic data for non-model organisms provides an avenue to answer several questions about the evolutionary trajectory and dispensability of DNA damage response (DDR) proteins. Protein sequence analyses, including those of *Nucleospora cyclopteri* sequenced herein, show that DDR and DNA replication proteins are, on average, two times longer than their housekeeping counterparts, regardless of organismal lifestyle or mitochondrial status. Furthermore, our data support the hypothesis that at least in some parasitic lineages, protein length compaction happened prior to the emergence of parasitic lifestyles. Finally, we show that in the analysed proteomes, the “modulation of nucleotide pools’ and Nucleotide Excision Repair pathway are the most preserved DDR pathways, with DPOLA and ERCC3 being the most preserved DDR proteins.

## Introduction

Eukaryotic cells endure thousands of DNA lesions each day (Jackson and Bartek 2009). Recognizing, signalling, and repairing DNA damage is therefore essential for cellular maintenance and for ensuring accurate transfer of DNA to daughter cells. The DNA damage response (DDR) is vital for the proper development and disease prevention of multicellular organisms. For example, in adaptive immunity, antibody diversification requires control of several DDR proteins (Sheppard et al. 2018; Cervantes-Gracia et al. 2021). In some unicellular parasites, such as *Trypanosoma brucei*, DDR proteins mediate host immune system evasion by changing their protective variant surface glycoprotein (VSG) coat. This process occurs close to telomeres and is facilitated by the presence of double-strand breaks (DSBs) in DNA (Horn and McCulloch 2010). Crucially, loss of specific DDR proteins such as those involved in DNA mismatch repair (MMR) can lead to hypermutations. The genomic variation that arises from these hypermutations in turn allows some unicellular organisms to evolve and exploit new habitats and rapidly adapt to new environmental stressors (Steenwyk et al. 2019; Phillips et al. 2021). Hypermutations could lead to genomic expansion or contraction accompanied by long or short protein-coding genes, respectively. The evolutionary trajectory towards genomic expansion or reduction is, however, dependent on fitness. Previous studies have shown that the evolutionary trajectory in the genomes of organisms with hypermutations is often biased towards reduction (Eisen and Hanawalt 1999; Steenwyk et al. 2019). DDR pathways are, therefore, critical for both multicellular and unicellular eukaryotes. However, these pathways are only well-studied in humans and a handful of model organisms, while various understudied eukaryotic lineages, for which genomic data exists, remain underexplored.

Protein-coding genes, encompassing those involved in DDR pathways, evolve through many mechanisms, such as shrinkages, expansions, base mutations, duplications, and fusions (Levinson and Gutman 1987; Björklund et al. 2006; Giacomelli et al. 2007; McDonald et al. 2011). It has been demonstrated that in most genomes, the genetic sequences of functional domains of these proteins are constrained with most of the nucleotide variation occurring in the interdomain regions (Kurland et al. 2007; Wang et al. 2011; Light et al. 2013), although exceptions have been documented in the genomes of obligate intracellular parasites (Nakjang et al. 2013).

With the rise of next-generation sequencing and the availability of raw data repositories, genomes of numerous eukaryotic organisms, especially those that are difficult to culture or those residing in various environmental niches, are now publicly accessible. It is captivating to discern how DDR pathway evolution aligns with the emergence of diverse lifestyles among different eukaryotic lineages. One such lifestyle is intranuclear parasitism. Even with their fascinating life strategy and the potential to exploit their host's DDR protein repertoire, the sequenced genomes of only a handful of intranuclear eukaryotes, namely, *Paramicrosporidium saccamoebae*, *Giardia* spp., and *Enterospora canceri* (Adam 2000 ; Corsaro et al. 2014; Wiredu Boakye et al. 2017), are currently publicly available. To this end, we have now sequenced the genome of *Nucleospora cyclopteri*, an intranuclear microsporidia that infects lumpfish (Freeman et al. 2013). We have also used phylogenomic and bioinformatic analyses to investigate the impact intranuclear parasitism, cytoplasmic parasitism, extracellular parasitism, and free-living lifestyles have on DDR protein length evolution. We analysed the homologues of 432 DDR proteins, 35 DNA replication proteins, 42 metabolic proteins, and 21 housekeeping proteins in the genomes of 67 eukaryotes. We used protein functional domain analyses to further investigate the exact sites of shrinkage or expansion in the homologue structures. We detailed which DDR proteins and pathways are preserved and to what degree they differ in protein sequence length and structure. With the inclusion of several genomes of intracellular parasites, known to have undergone genome compaction due to unique evolutionary pressures, these analyses shed light on the minimal DNA repair-ome. In addition, homologues of human DDR proteins that have undergone protein length compaction or expansion were revealed.

This study serves as a valuable resource for those keen on delving deeper into DDR pathways across a spectrum of eukaryotes, especially in the context of non-model organisms, and to understand minimal protein requirements and configurations essential for DDR pathways. In organisms such as the trypanosomatids and humans, DDR factors are often targets for drug development (Genois et al. 2014 ; Vieira-da-Rocha et al. 2019 ). A better understanding of their evolutionary trajectory could provide better drug targeting strategies and a better understanding of the fundamental biological mechanisms of DDR signalling.

## Results

### DPOLA and ERCC3 Are the Most Preserved DDR Proteins in Eukaryotes

**Fig. 1.**
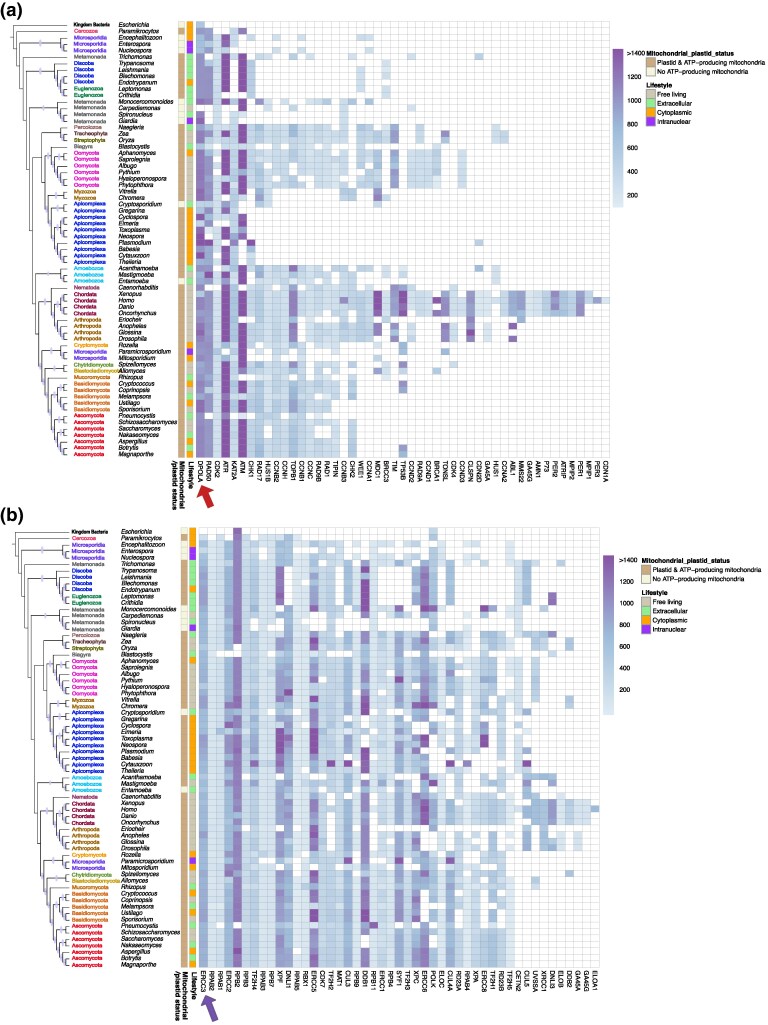
Profiling of DNA damage repair pathways. The heatmap shows the presence and length of constituent orthologues. Proteins that were not found to be encoded by the analysed genomes are represented by blank spaces. The phylogenetic positions in the cladogram are derived from maximum likelihood analyses performed on a concatenated alignment of 52 proteins. Oval symbols on the phylogenetic tree represent bootstrap support values for the corresponding nodes greater than 70. Proteins were grouped on the *x*-axis according to the DDR pathway they are part of a) *Checkpoint Factors*: Arrow pointing to DPOLA which is the most preserved DDR protein across the 68 organisms analysed in this study, including bacteria. b) *Nucleotide Excision Repair Pathway (NER)*: Arrow pointing to ERCC3 which is the most preserved DDR protein amongst the eukaryotes analysed here. Other pathways analysed in this study can be found in supplementary fig. S1–S21, Supplementary Material Online.

Although the proteomes used in this study were parsed and pathways classified for orthologues of known human DDR proteins, we found that 5 out of the 432 human DDR proteins (∼1%) did not have identifiable orthologues in model organisms, such as *Xenopus* and *Danio* spp. Our DDR pathway classification was performed according to previous work (Pearl et al. 2015). The DDR protein found to be encoded in all the proteomes used in our analysis, including that of *E. coli* (prokaryotic control proteome), was DPOLA (Arrow in Fig. 1a). The preservation of DPOLA is perhaps not surprising, as it is critically important for DNA synthesis (Starokadomskyy et al. 2016). ERCC3 was the only other protein, for which orthologues were identified in all eukaryotic organisms analysed in this study (Arrow in Fig. 1b).

### Modulation of Nucleotide Pools and NER are the Most Preserved DDR Pathways in Eukaryotes

**Fig. 2.**
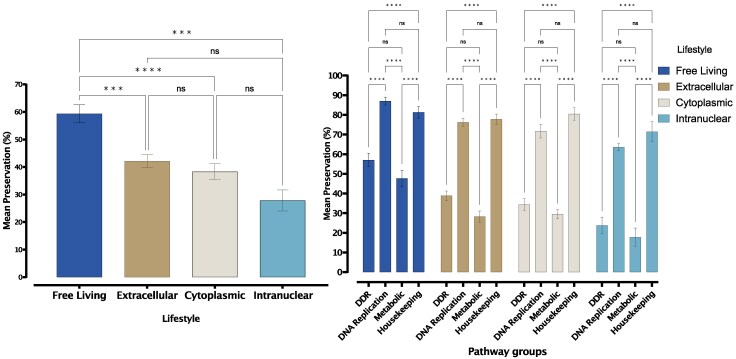
Mean preservation of DDR pathways: left) Analyses of the four lifestyles investigated show that there is a significant difference in overall mean preservation between lifestyles (one-way ANOVA, *F* (3, 64) = 13.08, *P* < 0.0001) with a gradual decline in preservation as organisms move from a free-living lifestyle to an intranuclear lifestyle. right) Mapping the preservation of DDR, DNA replication, metabolic, and housekeeping pathways against different lifestyles show that DNA replication and housekeeping pathways are more preserved across all lifestyles. Error bars represent the standard error of the mean.

None of the analysed eukaryotic proteomes contained orthologues of all 432 human DDR proteins. However, some DDR pathways had more constituent proteins in the analysed proteomes than others. For example, on average, 4 out of 6 proteins in the nucleotide pool modulation pathway had orthologues in the eukaryotic proteomes used in this study. In other words, on average, 65% of the proteins in the human nucleotide pool modulation pathway had orthologues encoded in the eukaryotic genomes analysed. We refer to this value as the preservation value for the pathway; supplementary table S2, Supplementary Material online lists the preservation values for each of the DDR pathways investigated in this study and shows that modulation of nucleotide pools and NER pathways are the most preserved in the eukaryotic genomes investigated. In general, there is a significant difference in the mean pathway preservation across the four lifestyles investigated (free-living, extracellular, cytoplasmic, and intranuclear [one-way ANOVA, *F* (3, 64) = 13.08, *P*  *<* 0.0001]). More specifically, pathways were generally more preserved in free-living organisms than in symbionts (Fig. 2, left). Among the organisms analysed, *Paramicrocytos*, *Giardia*, *Spironucleus*, *Enterospora*, and *Nucleospora* harboured the smallest eukaryotic DDR protein repertoires. Specifically, their proteomes contained only 66, 71, 71, 85, and 102 of the 432 human DDR proteins investigated, respectively. Our results indicate that DNA replication and housekeeping pathways are more preserved than metabolic and DDR pathways across all investigated lifestyles (Fig. 2, right). Moreover, lifestyle significantly influences pathway preservation, as evidenced by a notable interaction effect between lifestyle and the four pathway groups (two-way ANOVA, *F*(9, 256) = 2.293, *P* = 0.0172).

### Assessing the Impact of ROS-producing Organelles on Protein Length

**Fig. 3.**
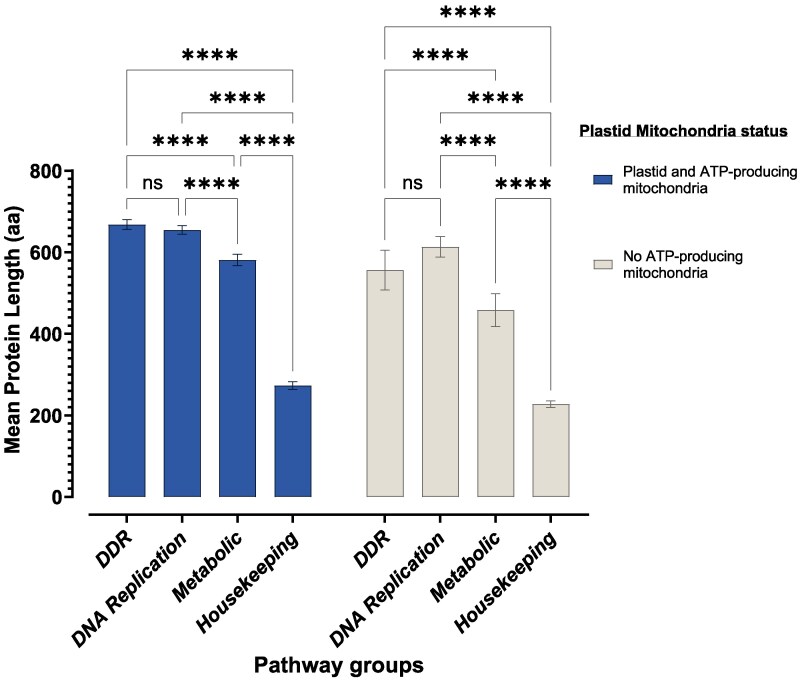
Comparing the length of DDR proteins in organisms with different plastid and mitochondrial status. The way that functional pathway groups (DDR, DNA replication, metabolic and housekeeping) influenced protein length did not depend on plastid/mitochondrial status (*P* = 0.1239). Functional pathway groups, however, significantly influenced protein length (*P* < 0.0001), with DDR and DNA replication proteins being consistently longer than their housekeeping counterparts across all plastid/mitochondria status groups.

We compared the protein lengths between organisms with plastid and ATP-producing mitochondria and no ATP-producing mitochondria to understand if the presence of reactive-oxygen-species-producing organelles, such as ATP-producing mitochondria and plastids, has an impact on protein size evolution (Fig. 3). The classification of organisms with ATP-producing mitochondria was based on Müller et al's classification (Classes 1–4 = organisms with ATP-producing mitochondria, Class 5 = organisms without ATP-producing mitochondria) (Müller et al. 2012). Our results show there was no significant effect of plastid/mitochondrial status on protein length. Furthermore, our data show that DDR and DNA replication proteins are longer compared to their housekeeping and metabolic counterparts in both organisms with plastid/ATP-producing mitochondria and no ATP-producing mitochondria. Moreover, the impact of plastid/mitochondrial status on protein length does not differ between DDR, DNA replication, metabolic, and housekeeping pathways (two-way ANOVA, F (3, 264) = 1.937, *P* = 0.1239) (Fig. 3).

### Protein Length Expansion Lies in Regions of Unknown Function

An unexpected observation from this study was that some proteomes contained proteins that were considerably longer than their human homologues. This included proteomes of unicellular organisms, such as *Endotrypanum*, *Neospora,* and *Plasmodium*. Typical examples of these unusually long proteins in unicellular organisms are MLH1, RUVB1, and ATR (MOLV88_240014700, XP_012897480.1 and KAH7831821.1). Due to the widespread misannotation of protein-coding genes in published genomes, often caused by the reliance of annotation programmes on gene models from related organisms that also contain errors, it is likely that the lengths of some of the extremely long proteins identified in our analyses have been affected by this issue. To address this, we systematically cross-referenced the predicted protein lengths with publicly available transcriptomic databases (e.g. EupathDB), focusing on the largest protein in the most conserved orthologue set in each pathway. Of the 20 proteins investigated, only one showed definite evidence of misannotation (UNG in *Blastocystis:* XP_012896248.1; see notes in supplementary table S1, Supplementary Material online). It is, however, unlikely that most of the proteins listed above represent errors in gene model predictions as there is transcriptomic evidence that covers the entire length of their predicted coding sequence in the EupathDB database or the gene model predictions were performed with mRNA sequencing data (see notes in supplementary table S1, Supplementary Material online).

We performed a Spearman's correlation test between protein length, interdomain length, and functional domain length to determine the hotspots for the observed protein length expansion. There was a very strong positive correlation between protein length and interdomain length (r(66) = 0.940, *P* < 0.0001). Similarly, there was a moderate correlation between protein length and functional domain length (*r*(66) = 0.464, *P* < 0.0001) and between Interdomain length and functional domain length (*r*(66) = 0.449, *P* < 0.0001). These results suggest that the increase in protein length in the pathways analysed is primarily due to the expansion of interdomain regions (supplementary fig. S24, Supplementary Material online). A one-way ANOVA test was performed to compare the effect of the four pathway groups on functional domain and interdomain length. This showed that there was a significant difference in mean functional domain length between all four pathway groups, with DNA replication proteins harbouring the longest functional domain regions (*P* < 0.0001) (supplementary fig. S25, Supplementary Material online). Analyses on interdomain length revealed that similarly, there was a significant difference in the length of interdomain regions between all four pathway groups analysed with DDR proteins and housekeeping proteins harbouring the longest and shortest interdomain regions, respectively (*P* < 0.0001) (supplementary fig. S25, Supplementary Material online). On average, DDR and DNA replication proteins are approximately 84% longer than housekeeping proteins and 24% longer than metabolic proteins.

### Analysis of DDR, DNA Replication, Metabolic and Housekeeping Protein Lengths Across Eukaryotes With Different Lifestyles

**Fig. 4.**
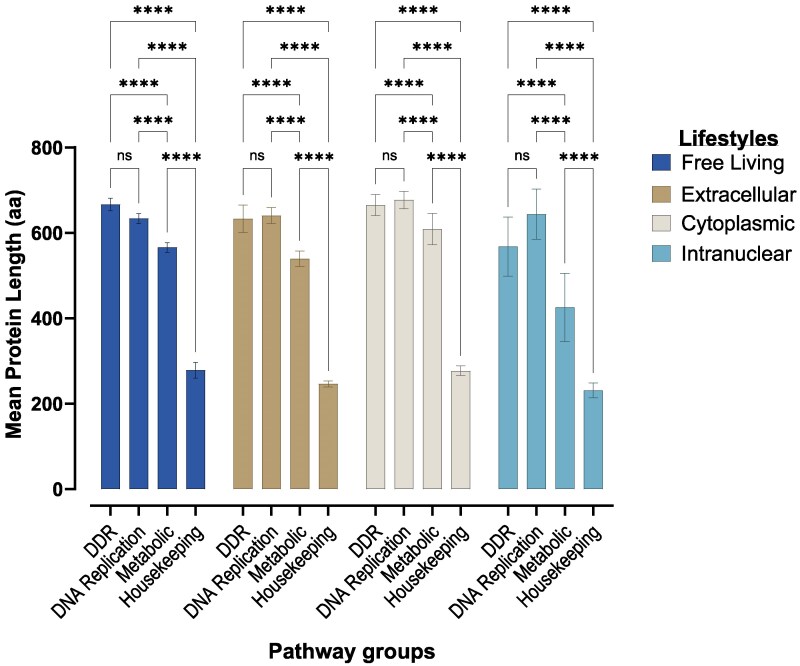
Assessing the effect of lifestyle on the length of proteins in DDR, DNA replication, metabolic, and housekeeping pathways. Proteins belonging to DDR and DNA replication pathways are longer than those in metabolic and housekeeping pathways, regardless of the organism's lifestyle (*P* < 0.0001). Error bars represent the standard error of the mean.

Our analyses revealed that DDR and DNA replication proteins are longer than metabolic and housekeeping proteins, regardless of the organism's lifestyle (Fig. 4). The effect that these four pathway groups have on the protein's length does not differ between lifestyles (two-way ANOVA, *F*(9,256) = 0.9415, *P* = 0.4896).

### Genome Statistics of Nucleospora Cyclopteri

The nuclear genome of *Nucleospora cyclopteri* assembled in this study is 4.76 Mb in size spanning across 1,209 scaffolds. It contains 2,939 predicted genes. The GC content is 26.3%, which is within the range of previously sequenced Microsporidia belonging to the family Enterocytozoonidae (22.44—40.15%; Wiredu Boakye et al. 2017 [Table 1]).

**Table 1:** Comparing the genome assembly statistics for *Nucleospora cyclopteri* with other members of the Enterocytozoonidae family

	*N. cyclopteri*	*H. eriocheir*	*H. eriocheir canceri*	*E. canceri*	*E. hepatopenaei*
**Assembly Size (Mb)**	4.76	4.57	4.84	3.10	3.26
**GC %**	26.3	22.44	23.16	40.15	25.45
**Number of contigs**	1209	1300	2344	537	64
**N_50_**	6279	17,583	3349	11,128	125,008
**# genes**	2681	2716	3058	2179	2540
**Coverage (X)**	920	4477.89	63.18	288	363

### Comparison of DDR Proteins With Extreme Lengths Using Structure Prediction

To get a visual representation of how interdomain protein extensions and/or deletions affect tertiary protein structures, we compared experimentally determined- or predicted structures of the human or Saccharomyces cerevisiae homologue, with the homology-modelled structures of the smallest and the longest homologues. As expected, in most cases, the homology models of the longest proteins correspond only to the part of their human or yeast homologues, whose structure is available or can be modelled from structures of orthologues in other species. However, in some cases, additional 3D folds were indicated in the extended regions. Therefore, we predicted structures of the longest orthologues of 11 selected proteins using AlphaFold 3 (Abramson et al. 2024) and used the models to search multiple structural and domain databases for structural similarity search by FoldSeek (van Kempen et al. 2024). The approach showed that 6 out of 11 proteins tested are predicted to contain additional domains, such as pleckstrin-homology (TYDP1), helicase (PIF1), ATPase (MRE11), or helical repeat (PARP1, TOP1) domains, in their extensions, indicating possibly extended interaction networks or functions. The remaining proteins exhibit mostly multiple insertions without any predicted folds. The predicted structures of the shortest proteins consistently corresponded to the representatives from model species, but typically lacked long stretches of amino acids that encode functional domains in their human or yeast homologues (supplementary table S5, Supplementary Material online).

## Discussion

### Proteins Involved in DNA Replication Pathways are Preserved Even in Extreme Parasites

DNA replication stood out as the most preserved pathway amongst the four pathway groups in our analyses. Understandably, transcription and DNA replication for both eukaryotes and prokaryotes is indispensable, and so the retention of proteins involved in this pathway across our analysed proteomes should not be surprising (Samson and Bell 2016). However, considering some of the proteomes analysed here belong to extreme parasites such as microsporidians that have undergone extreme gene loss, it was interesting to see that even in these parasites, DNA replication pathway conservation remained high. Amongst DDR pathways, modulation of nucleotide pools and nucleotide excision repair pathways (NER) were the most preserved. This was expected given that proteins involved in modulation of nucleotide pools, such as Ribonucleoside-diphosphate reductase (RIR), RIR1 and RIR2, are responsible for de novo dNTP synthesis and for maintaining balanced nucleotide pools (Kunz et al. 1994). Despite the critical nature of DNA synthesis for life, the proteomes of *Spironucleus, Entamoeba*, and *Giardia* did not appear to contain any ortholog of the human RIR proteins (Also known as type I RNR proteins). This observation is consistent with the literature suggesting that their absence is an adaptation to extreme parasitism. *Giardia*, for instance, might have developed specialised nucleotide transporters to syphon nucleotides from its host (Baum et al. 1989; Adam 2001; Loftus et al. 2005; Xu et al. 2014). We also found that the proteome of *Monocercomonoides* did not contain noticeable RIR proteins. Instead, the proteome of this amitochondriate flagellate contained four nucleoside triphosphate proteins, which are believed to substitute the function of RIR proteins in de novo nucleotide synthesis and regulation (Karnkowska et al. 2019). This is peculiar as ribonucleoside triphosphate reductase proteins, also referred to as type III RNRs, are typically found in bacteria and archaea (Fontecave et al. 2002). BLAST search results suggest these proteins may have been horizontally acquired, but further phylogenetic analyses are required to confirm this hypothesis. Similarly, the proteome of *Blastocystis* did not contain any homologues of the RIR proteins. Instead, it contained orthologues of the type III RNRs. Considering type III RNRs are inactivated by oxygen, it is possible that these organisms, together with the other eight organisms investigated in this analyses that contained at least one RIR protein as well as type III RNRs (*Aphanomyces*, *Acanthamoeba*, *Phytophthora*, *Naegleria*, *Allomyces*, *Pythium,* and *Saprolegnia*), may use this protein during their anaerobic life stages. The list of type III RNRs found in the above-mentioned organisms can be found in supplementary table S3, Supplementary Material online. Although *Trichomonas* appeared not to possess type I RNR proteins, there is evidence to support the presence of type II RNR proteins in the *Trichomonas* proteome that has either archaeal or eukaryal origins (Lundin et al. 2010). In line with published data that suggest a reduced repertoire of DDR proteins in *Carpediemonas*, we observed that it lacked any type of RNRs in its proteome (Salas-Leiva et al. 2021). This is particularly intriguing as *Carpediemonas* is a free-living organism and cannot benefit from syphoning host nucleotides as some closely related parasites in the Metamonada lineage do (Karnkowska et al. 2019). It is therefore still unclear how or where *Carpediemonas* gets its nucleotides from for DNA replication and repair.

Nucleotide excision repair (NER) is one of the principal DNA repair pathways, responsible for removing a wide range of DNA lesions typically induced by exogenous chemical agents or UV irradiation. NER is subdivided into two subpathways: global genome repair (GG-NER), which operates throughout the genome, and transcription-coupled repair (TC-NER), which specifically removes lesions from the transcribed strand of actively transcribed genes. Xeroderma pigmentosum group C-complementing protein (XPC) is a core protein required for GG-NER, whereas ERCC6 and ERCC8 are essential for TC-NER (Balajee and Bohr 2000). Given NER's critical role in repairing UV-induced damage, the patchy preservation of its core proteins across the proteomes analysed was initially surprising. However, this observation aligns with previous studies. For instance, Sekelsky and colleagues have previously reported the absence of core TC-NER proteins in *Drosophila* and other arthropods (Sekelsky et al. 2000). Recent studies using excision repair sequencing and genome-wide repair mapping have highlighted the versatility of XPC, demonstrating that XPC could function in both GG- and TC-NER in Drosophila. This suggests that, in closely related arthropods lacking canonical TC-NER proteins, XPC may compensate for the absence of canonical TC-NER proteins (Deger et al. 2022). The absence of core NER proteins in members of the Basidiomycota fungal clade has also been previously documented. In these fungi, alternative excision repair pathways and photoreactivation are believed to substitute canonical NER pathway function (Wong et al. 2019). Notably, *Giardia*, *Spironucleus*, *Enterospora*, and *Paramikrocytos* were the only organisms in our dataset lacking proteins from both canonical NER subpathways. This finding is corroborated by previous studies for *Paramikrocytos*, *Giardia*, and *Spironucleus* (Adam 2000; Feltrin et al. 2020; Onuț-Brännström et al. 2023). Although one previous study predicted the presence of NER core proteins in *Giardia* and *Spironucleus* (Karnkowska et al. 2019), our BLAST searches using NER proteins such as ERCC6 from the closely related metamonad *Monocercomonoides* resulted in low query coverage and low sequence identity (<30%), casting doubt over the existence of functional NER proteins in these organisms. It remains unclear if, and how, parasites such as *Giardia* and *Spironucleus*, which have an environmental life cycle stage, repair DNA damage induced by UV irradiation. Finally, the complete absence of all core NER proteins in the microsporidian *Enterospora* is particularly intriguing, given that other closely related microsporidians, such as *Nucleospora*, encode at least one of these proteins. While the possibility of incomplete genome coverage or annotation errors cannot be ruled out, the apparent lack of most NER proteins in *Enterospora* strongly suggests a genuine loss of this pathway, which warrants further investigation.

Homologous recombination (HR) and nonhomologous end joining (NHEJ) are fundamental biological processes essential for the repair of double-strand DNA breaks. RAD51 and its out-paralogues (RAD51B, RAD51C, RAD51D, XRCC2, and XRCC3) are key regulators of HR in eukaryotes; their absence is particularly lethal in higher eukaryotes but not in other eukaryotic lineages (Bleuyard et al. 2005; Markmann-Mulisch et al. 2007). Nonetheless, our analyses show that nearly all of the eukaryotic proteomes examined contain RAD51. In those few lineages lacking RAD51, such as *Phytophthora*, *Melamspora*, *Aspergillus*, Metamonda, and Euglenozoa (see supplementary fig. S15, Supplementary Material online), we identified either a homologue of one of its out-paralogues or DMC1, a protein with considerable sequence similarity and chemical properties to RAD51 (Lan et al. 2020; Steinfeld et al. 2019). This finding suggests that the HR pathway is likely conserved across these diverse organisms. We find that core NHEJ proteins such as DNLI4, XRCC5, and XRCC6 (KU70/80) are absent in lineages in our analyses with parasitic lifestyles (supplementary fig. S12, Supplementary Material online). Considering the level of functional overlap between HR and NHEJ, it is plausible that genome-streamlining pressures associated with parasitic lifestyles led to the loss of the NHEJ machinery. A more comprehensive exploration of the NHEJ evolution in parasites can be found in Nenarokova et al. (2019).

The evolutionary conservation of certain proteins categorized within DDR pathways, such as POLA1, ERCC3, TOP2B, among others, likely reflects their fundamental roles in cellular processes such as DNA replication and transcriptional regulation, rather than their auxiliary roles in DDR. We recognize that these proteins might be highly conserved for reasons beyond their involvement in DDR pathways. However, we chose to adhere to the genome-wide classification established by Pearl et al. (2015) for consistency and comparability across past studies. We do, however, acknowledge that these categorisations may not perfectly capture the primary biological functions of such proteins. Future efforts to refine DDR protein classifications could improve the specificity of pathway assignments and facilitate a more nuanced understanding of their evolutionary conservation.

### Absence of an Intrinsic Energy Source May Have Led to the Evolution of Smaller Protein Homologues in Eukaryotes Without ATP-Producing Mitochondria

Poorly preserved pathways and shorter proteins are well-known adaptations of parasitism (Williams et al. 2002; Beznoussenko et al. 2007; Corradi et al. 2010; Keeling et al. 2010 ). Our analyses showed that not all parasites had poorly preserved pathways and short proteins, suggesting that parasitism is not always accompanied by a lack of pathway preservation and gene compaction culminating in shorter proteins; corroborating conclusions from Butenko et al. (2020) and Salas-Leiva et al (2021). Exploring the influence of ROS-producing organelles on protein length across distantly related parasites is challenging, as this effect can be masked by the distinct evolutionary pressures acting on each species. However, the microsporidians’ parasitic lifestyle, coupled with the presence of both species that possess ATP-producing mitochondria and those that lack them in this lineage, presents a unique opportunity to investigate the relationship between parasitism and the influence of ROS-producing organelles on protein length. *Paramicrosporidia*, a microsporidian with an ATP-producing mitochondria, encode much longer proteins as compared to other members of the phylum Microsporidia analysed here that do not possess ATP-producing mitochondria (*Encephalitozoon, Enterospora*, and *Nucleospora*) (supplementary fig. S22, Supplementary Material online). The absence of intrinsic ATP generation through oxidative phosphorylation is likely to have posed a bioenergetic constraint that ultimately fostered the evolution of smaller genes and, hence, shorter proteins in microsporidians, as smaller genes are less energetically expensive to maintain, express, and replicate. Conversely, it is generally accepted that the ATP-rich environment provided by a functional mitochondrion fosters the evolution of larger genes that, in turn, encode longer proteins (Lane and Martin 2010; Lane 2011). However, intracellular abundance of ATP alone is unlikely to be the driving force behind the evolution of longer proteins (Lynch and Marinov 2015). Longer proteins must confer an adaptive advantage, especially when occurring in extremely compacted genomes such as those of extreme parasites. Here, we speculate that the presence of ROS-producing organelles, such as ATP-producing mitochondria, increases the selective pressure for the evolution of longer proteins. Considering the deleterious effects of ROS on protein stability (Zuo et al. 2015), it is possible that the amino acids in regions of low complexity of the elongated proteins are used to shield functional domains from the harmful effects of ROS. It has been demonstrated that protein sequence elongation at the C- or N-terminal can confer extra stability to the protein (Matsuura et al. 1999; Liu et al. 2001). Interestingly, several of the extensions observed in the very long homologues analysed here were in the C- or N-terminal of the core protein (supplementary table S5, Supplementary Material online). Furthermore, low complexity regions, such as those featured in the very long proteins in this study, have long been known to have mechanistic functions. Some of these include interaction with phospholipids (Robison et al. 2016), coordinating metal ions (Zhu and Karlin 1996), and having adhesive properties (Haritos et al. 2010; So et al. 2016). A future experiment to test this hypothesis would be to expose homologous proteins with extreme lengths (i.e. a very long homologue and a very short homologue) to oxidative agents and measure the rate of protein denaturation. As genomes of obligate parasites with functional mitochondria that are closely related to parasites without functional mitochondria become publicly available, this hypothesis can be further explored.

### Length Variation Between DDR, DNA Replication, Metabolic and Housekeeping Proteins Suggest They Are Influenced by Different Evolutionary Pressures

Proteins involved in the DNA replication process are intimately involved in several DDR processes. As such, it was not surprising to find that there was no difference in length between proteins in these two pathway groups. Our results, however, showed that DDR and DNA repair proteins were consistently longer than metabolic and housekeeping proteins (Fig. 4). It is not clear from our analyses why DDR and DNA repair proteins would evolve to be larger than their metabolic and housekeeping counterparts. However, previous studies have suggested a positive correlation between protein size and expression profiles (Warringer and Blomberg 2006; Moutinho et al. 2019). Thus, highly expressed proteins, such as metabolic and housekeeping proteins, may experience evolutionary pressure to undergo shrinkage to make translation and post-translational folding more energy-efficient (Kim et al. 2014; Uhlen et al. 2015; von Stechow and Olsen 2017). Evidence from transcriptomic analyses from several organisms corroborates this hypothesis as genes involved in DNA repair pathways are not as heavily transcribed as their metabolic counterparts (Ohtsu et al. 2007; Shin et al. 2018; Callejas-Hernández et al. 2019; Mardanov et al. 2020; Fan et al. 2022).

**Fig. 5.**
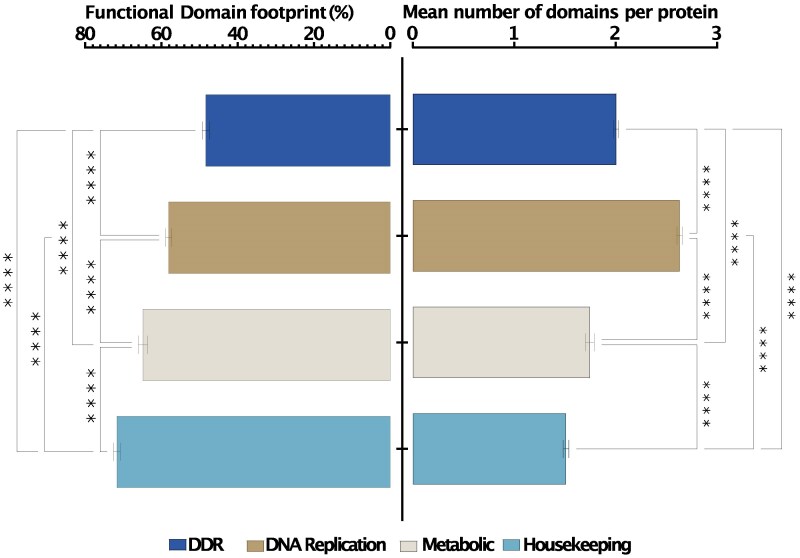
Assessing the footprint of functional domains between proteins in DDR, DNA replication, metabolic, and housekeeping pathways. DDR and DNA replication proteins have relatively small functional domain footprints but pack a higher density of functional domains within those compact spaces compared to metabolic and housekeeping proteins.

Furthermore, longer proteins are hypothesized to evolve in response to a heightened need for protein–protein interactions within the cell (Cavalier-Smith 2005). In order to assess this hypothesis in relation to our data, we performed searches for 54 representative DDR and housekeeping proteins on the STRING protein–protein interaction database (Szklarczyk et al. 2021). We found that on average, a DDR protein was projected to have 15 physical interactions, while a housekeeping protein only had 6 (supplementary fig. S23, Supplementary Material online). This underscores the possible role of expanded interdomain regions in DDR proteins in aiding their increased protein–protein interactions. We further found that while DNA repair proteins are typically longer than metabolic and housekeeping proteins (Fig. 4) (*P* < 0.0001), domains of DNA repair proteins occupy a relatively smaller footprint compared to their metabolic and housekeeping counterparts (where footprint is the length covered by the functional domain relative to the length of the protein). Within that smaller domain footprint, DDR proteins often pack more individual functional domains (Fig. 5). That is, the increased length of DDR proteins for the most part is not due to the inclusion of multiple functional domains or expansion of single functional domains but actually due to the expansion of the interdomain space. The packaging of multiple functional domains within a small footprint further speaks to the involvement of DDR proteins in multiple interactions and the importance of the interdomain regions in aiding this function. This pattern appears to have no dependence on organismal lifestyle or mitochondrial status. In-paralogues can serve as an evolutionary sandbox for the birth of novel functional proteins. During speciation, these duplicated genes may accumulate mutations that expand or reduce the length of the proteins they encode (McClune and Laub 2020). While we observed notable differences in protein length among certain in-paralogues in the proteomes analysed, the longest proteins in the pathways we examined were generally single-copy with the exception of ARI1A in *Anopheles*. However, the difference in length between the paralogues of ARI1A in *Anopheles* was relatively small. As such, it is unlikely that the extreme lengths in protein sizes observed in some of the unicellular organisms investigated here are a result of the genetic plasticity that paralogues have.

### Intranuclear Lifestyle Is Not Accompanied by Significant Protein Length Reduction

Our analyses contained the only three published genomes of intranuclear eukaryotes, as well as a genome of a fourth intranuclear microsporidia we have sequenced for the first time as part of this study. We did not observe any correlation between intranuclear lifestyle and protein size. Furthermore, our ortholog clustering did not identify any protein families that were unique to all four intranuclear parasites, and our protein length analyses did not identify any unique patterns for this group of organisms, leaving the question of how these parasites are adapted to their unique lifestyle unanswered.

## Materials and Methods

### Sampling Wild Lumpfish and Purification of *Nucleospora cyclopteri* Spores

Atlantic lumpfish (*Cyclopterus lumpus*) were sampled from a commercial fishing boat operating from the coast of Iceland in December 2016. During necropsy, kidneys with advanced clinical signs of *Nucleospora* infections were crushed with a sterile pestle and mortar in 1 × PBS. The homogenate was then filtered through a 100-μm mesh followed by cell sieving through 40-μm filter to remove tissue debris, and the filtrate was further purified using a Percoll (Sigma) density gradient centrifugation (Taupin et al. 2006).

### Genomic DNA Extraction and Sequencing Protocols

Purified spores were subjected to bead beating followed by phenol/chloroform extraction and ethanol precipitation as previously described (Campbell et al. 2013). *Nucleospora cyclopteri* genomic DNA was used to generate a SPriworks fragment library (Beckman Coulter), which was sequenced using the MiSeq v. 2 platform at the University of Exeter Sequencing Service.

### Genome Assembly and Annotation

A total of 39,581,310 raw Illumina paired-end reads with a length of 250 bp were used in the analysis. These reads are deposited in the NCBI SRA database under the accession number SUB12014904. PRINSEQ (Schmieder and Edwards 2011) was used to filter and trim reads identified to have poor quality scores by FASTQC (Andrews 2010). This resulted in 32,709,299 paired-end reads with an average length of 139 bp. These reads were used for the assembly of the *N. cyclopteri* genome with Spades v. 3 (Prjibelski et al. 2020). The sparsity of introns in the genomes of most microsporidians make fast prokaryotic genome annotation tools ideal for their annotation, as has been done in previous microsporidian sequencing studies (Wiredu Boakye et al. 2017). As such, open-reading frames (ORFs), tRNAs and rRNAs of the *N. cyclopteri* genome were predicted by Prokka v. 1.11 (Seemann 2014). Further BLASTP and BLASTX searches were used to annotate predicted ORFs. Contaminating bacterial sequences, found in the initially assembled scaffolds, were removed using a combination of both BLASTP and BLASTX searches for putative ORFs and by BLASTN searches for rRNAs against the NCBI non-redundant protein (nr) and nucleotide (nt) databases, respectively. All BLAST searches were performed using default parameters.

### Identification of DDR, DNA Replication, Metabolic, and Housekeeping Orthologues

In this study, the predicted proteins of *N. cyclopteri* and those of 67 other organisms, whose proteomes are publicly available (supplementary table S4, Supplementary Material online), were parsed to identify homologues of DDR proteins. To accomplish this, the predicted proteins encoded by the genomes of these organisms were downloaded from NCBI (Agarwala et al. 2018), Ensembl (Zerbino et al. 2018), EupathDB (Aurrecoechea et al. 2017), or GigaDB (Sneddon et al. 2012) (FASTA files with protein sequences used in this study can be found here: https://drive.google.com/file/d/1q25rde-9vHaZBANvGSbq4bbV5PGl3LSg/view?usp=sharing). These protein sequences were grouped into orthologue families by running Orthofinder (V2.5.5) (Emms and Kelly 2019) with default parameters. Orthofinder assigned 995882 genes (90.1%) to 84761 orthogroups, which suggested our species sampling was good. The Orthofinder orthogroup output folder for human proteins was then parsed for 432 DDR, 35 DNA replication, 38 metabolic, and 21 housekeeping orthologue families. Although Orthofinder reliably detects orthologues in closely related species, its phylogeny-based approach can miss orthologues in distantly related lineages. To address this limitation, we supplemented our orthofinder analysis with orthology clustering data from InParanoiDB. Unlike Orthofinder, InParanoiDB identifies orthologues by comparing pairwise similarity scores for Pfam-predicted domains between pairs of reference proteomes (e.g. between human and yeast), thereby circumventing some of the challenges encountered by phylogeny-centric tools. Following the orthofinder analyses, we constructed a phylogenetic tree using the concatenated sequences of a set of shared proteins between the 68 organisms analysed, enabling us to define broad taxonomic clades. For each clade, we designated a reference organism present in InParanoiDB (e.g. Saccharomyces for Fungi). When Orthofinder failed to detect an orthologue for a given species, we first queried the corresponding human orthologue against the clade's reference organism proteome in InParanoiDB. If the human protein had a confirmed orthologue in the reference proteome, we then used that reference orthologue as “bait” to locate the corresponding orthologue of the query species in the Orthofinder pairing file between the reference organism and the query species. A flow chart explaining our orthologue clustering methodology can be found in supplementary fig. S26, Supplementary Material online. The DDR proteins used in this analysis were those used in Perl et al. (2015). The DNA replication proteins were those listed in the KEGG human DNA replication repository (Kanehisa et al. 2023), whereas the housekeeping proteins included those in Joshi et al. (2022) and Barta et al. (2023). Subsequently, the protein lengths for the predicted orthologues were extracted. The script used for the orthology clustering and extracting protein lengths can be found here: https://github.com/DrDomUoE/DDR_paper.git. To evaluate the performance of our orthologue-calling approach, we focused on orthologue groups that showed >95% conservation across the species analysed. Because these groups are so widely preserved, we reasoned that any reported absences are likely to be due either to methodological errors or rare instances of true loss. For any orthologues found to be missing within these groups, we manually investigated them through BLAST and literature searches to determine whether they could be detected in closely related species not included in our analyses. This enabled us to distinguish genuine biological absences from those potentially attributable to failure of our orthologue-calling, incomplete genome sequencing, or poor gene calling models. We identified 34 orthologue groups that were at least 95% conserved across all species examined. We next quantified the performance of our orthologue-calling method by counting the number of absences that we predicted to be due to methodological failure. Our estimates indicate that, for each orthologue group, our ortholog calling method successfully identified orthologues in 65 to 67 of the 67 species analysed (i.e. 95.5% to 100% coverage). Based on these figures, the probability of observing the recorded number of missing orthologues purely as a result of orthologue-calling failure (rather than genuine biological absences) was calculated to be between 0% and 17%. During these analyses, it became apparent that the *Eriocheir* proteome used here was severely incomplete due to partial coverage of the published genome assembly. Consequently, protein absences in this genome were excluded from our analysis.

### Phylogenetic Tree Construction

For the above-mentioned phylogenetic tree, Fifty-two single-copy othologues from the Orthofinder output were used to build a concatenated alignment for the construction of a species tree. More specifically, the proteins were initially aligned using MUSCLE v. 5.1 (Edgar 2004) with default parameters, trimmed with TrimAl v1.5.rev0 (Capella-Gutierrez et al. 2009) with default parameters. To identify the most appropriate protein model for each alignment, we used iqtree (v2.3.6) with the -m MF option (Minh et al. 2020). We used the concatenate option in Mega to concatenate the alignments from the ortholog families into a single file. We then used the predicted top protein models assigned by iqtree and the alignment length for each orthologue family to create a tabular formatted partition file as prescribed by the RaxML manual. The final concatenated alignment was passed to RaxML(8.2.12) (Stamatakis 2014) to construct a species tree with the following parameters: PTHREADS -T 20 -q, -m PROTGAMMAAUTO, -f a, -# 100, -x 12345, -*P* 54321.

### Functional Domain Prediction

Functional domain calling was accomplished by using pfam_scan.pl (Mistry et al. 2007) to query all proteins against a locally installed version of the Pfam-A.hmm database, which was downloaded on the 23 August 2024 onto a local server. This was run with default parameters (Eddy 2011). The number and length of the domains predicted for each protein were extracted from the pfam_scan.pl output file. The bash script we created for parsing the pfam_scan.pl output can be found online: https://github.com/DrDomUoE/DDR_paper.git.

### Prediction of Protein Structures

Structures of selected DDR proteins were homology-modelled using SWISS-MODEL (Waterhouse et al. 2018) and Phyre2 (Kelley et al. 2015) online web toolkits. De novo structure prediction was performed using AlphaFold 3 (Abramson et al. 2024). FoldSeek (van Kempen et al. 2024) was used to search for structurally similar proteins and domains in all available databases, including AlphaFold, PDB, and CATH. The predicted structures were superimposed with experimentally determined structures of respective human or *S. cerevisiae* homologues using the MatchMaker tool of UCSF Chimera (Pettersen et al. 2004).

## Supplementary Material

evaf089_Supplementary_Data

## Data Availability

The Whole Genome Shotgun project has been deposited at DDBJ/ENA/GenBank under the accession JASPEO000000000. The version described in this article is version JASPEO010000000. In house scripts and datafiles used in this study can be found online: https://github.com/DrDomUoE/DDR_paper.git
