# Supplementary material for: Evolutionary Insights into the Length Variation of DNA Damage Response Proteins Across Eukaryotes
Source: Genome Biol Evol. 2025 May 19;17(6):evaf089. doi: 10.1093/gbe/evaf089 (PMC12134460; doi:10.1093/gbe/evaf089)
Supplement: evaf089_Supplementary_Data [file evaf089_supplementary_data.zip › Supplementary Figures.pdf]

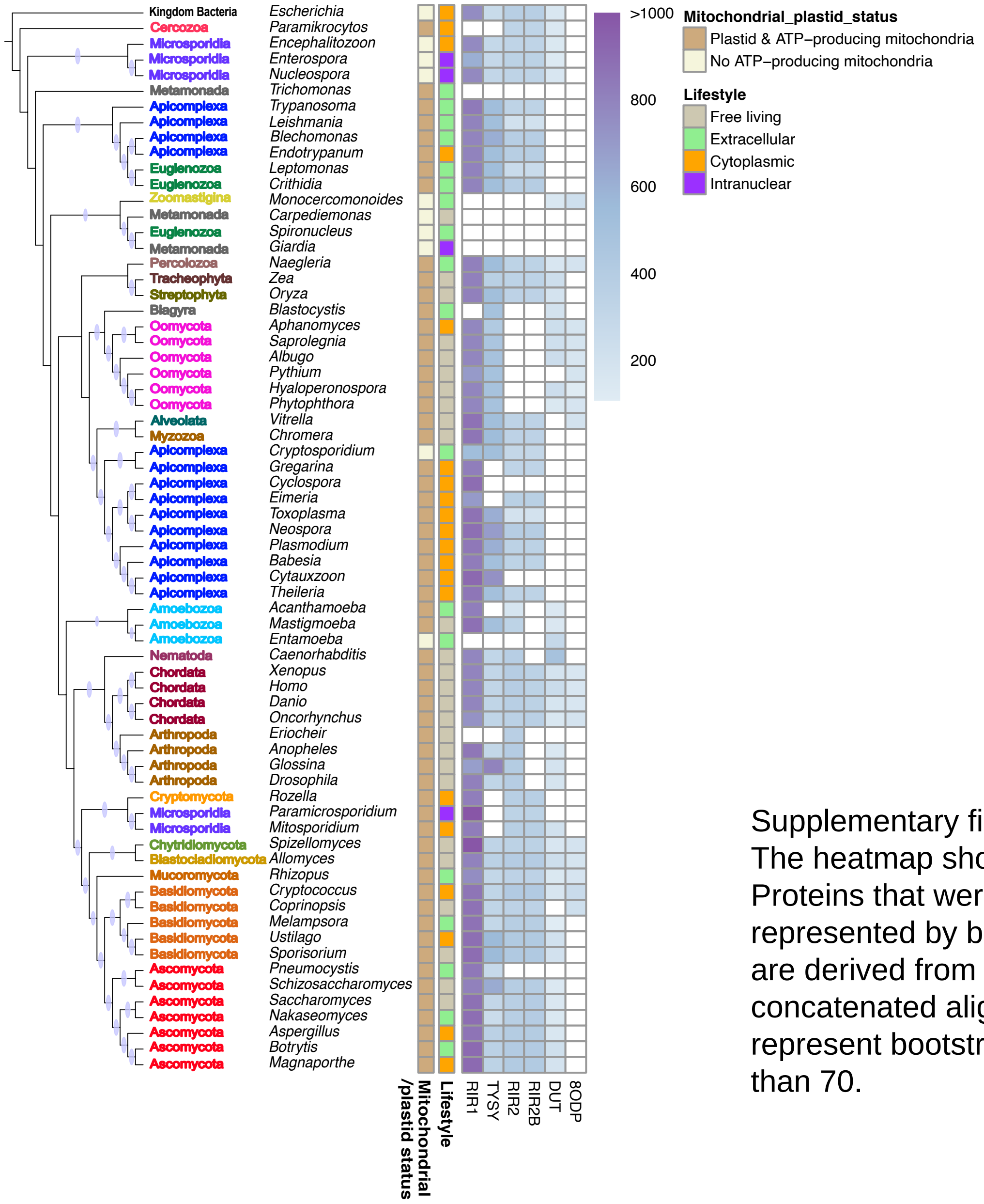

Supplementary fig 1: Profiling of nucleotide pools modulation pathways. The heatmap shows the presence and length of constituent orthologs. Proteins that were not found to be encoded by the analysed genomes are represented by blank spaces. The phylogenetic positions in the cladogram are derived from maximum likelihood analyses performed on a concatenated alignment of 52 proteins. Oval symbols on phylogenetic tree represent bootstrap support values for the corresponding nodes greater than 70.

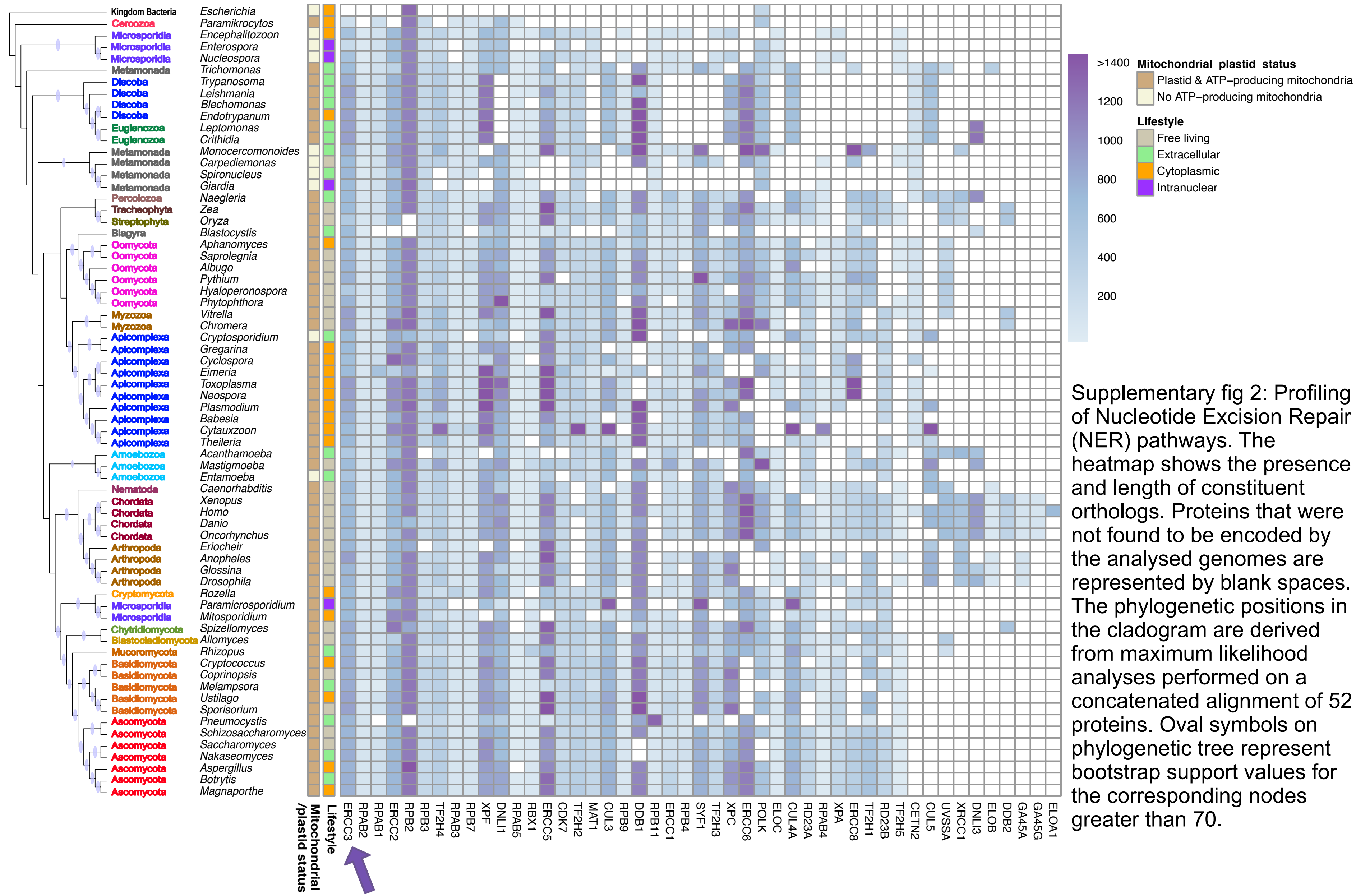

Supplementary fig 2: Profiling of Nucleotide Excision Repair (NER) pathways. The heatmap shows the presence and length of constituent orthologs. Proteins that were not found to be encoded by the analysed genomes are represented by blank spaces. The phylogenetic positions in the cladogram are derived from maximum likelihood analyses performed on a concatenated alignment of 52 proteins. Oval symbols on phylogenetic tree represent bootstrap support values for the corresponding nodes greater than 70.

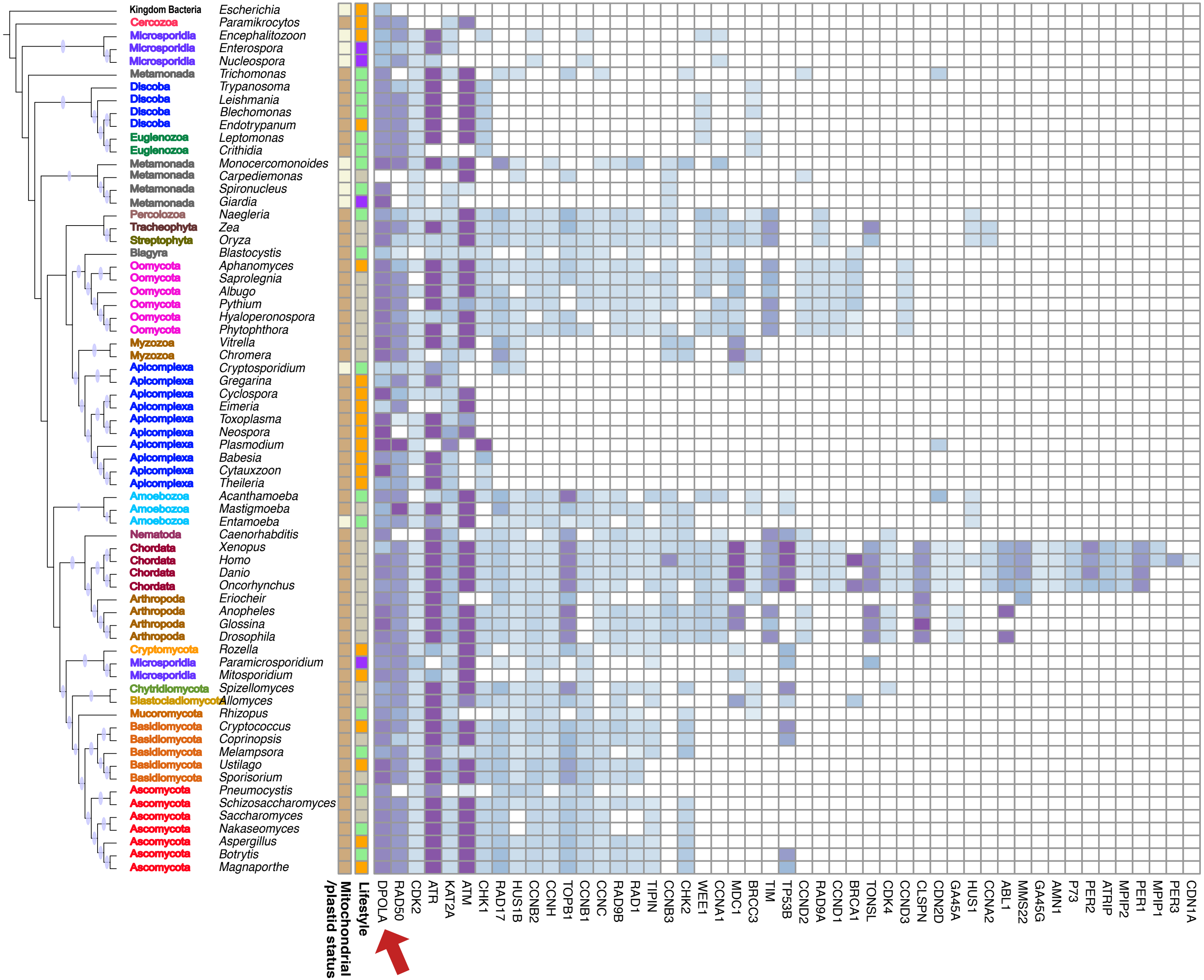

Supplementary fig 3: Profiling of Checkpoint factor pathways. The heatmap shows the presence and length of constituent orthologs. Proteins that were not found to be encoded by the analysed genomes are represented by blank spaces. The phylogenetic positions in the cladogram are derived from maximum likelihood analyses performed on a concatenated alignment of 52 proteins. Oval symbols on phylogenetic tree represent bootstrap support values for the corresponding nodes greater than 70.

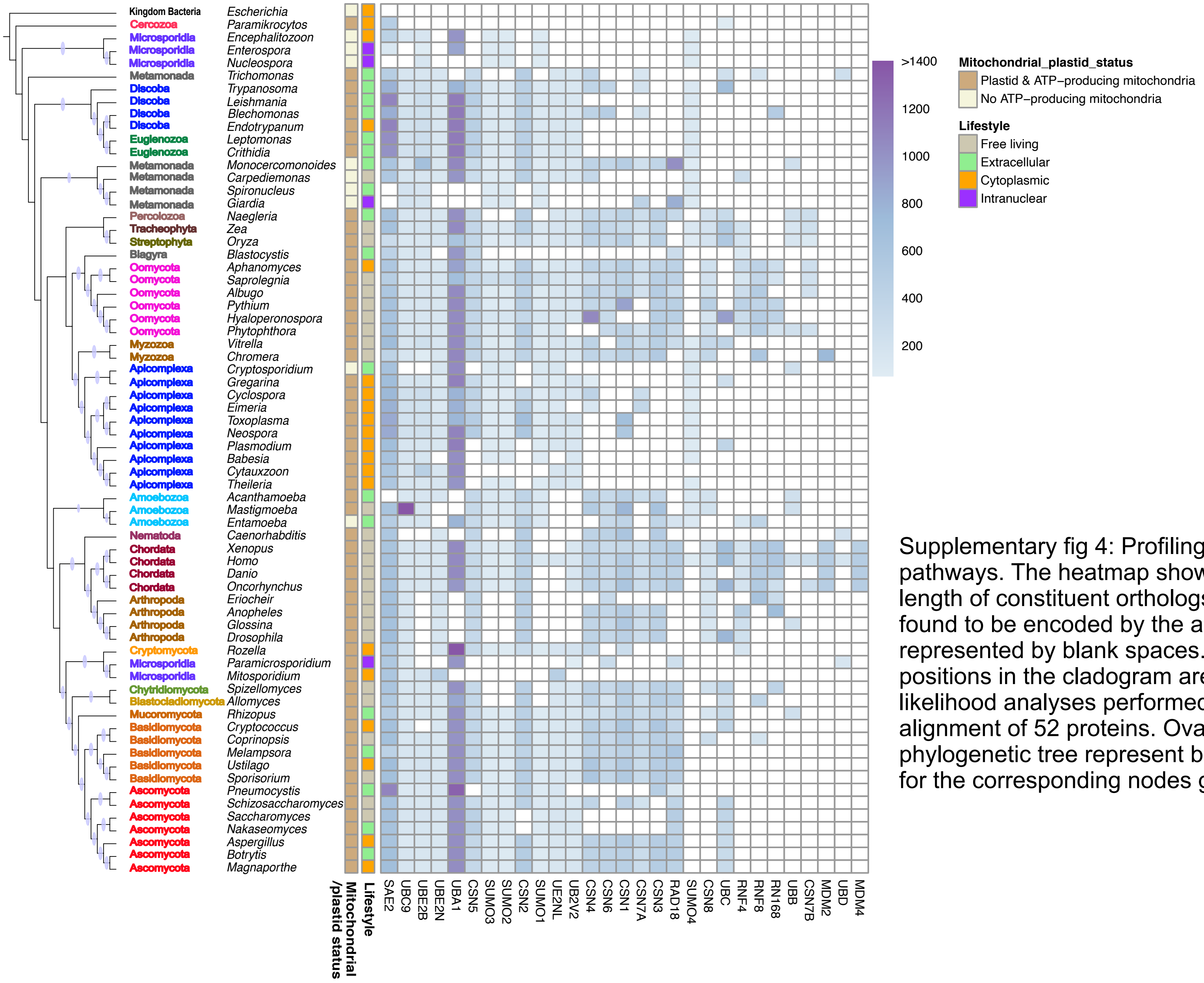

Supplementary fig 4: Profiling of ubiquitin response pathways. The heatmap shows the presence and length of constituent orthologs. Proteins that were not found to be encoded by the analysed genomes are represented by blank spaces. The phylogenetic positions in the cladogram are derived from maximum likelihood analyses performed on a concatenated alignment of 52 proteins. Oval symbols on phylogenetic tree represent bootstrap support values for the corresponding nodes greater than 70.

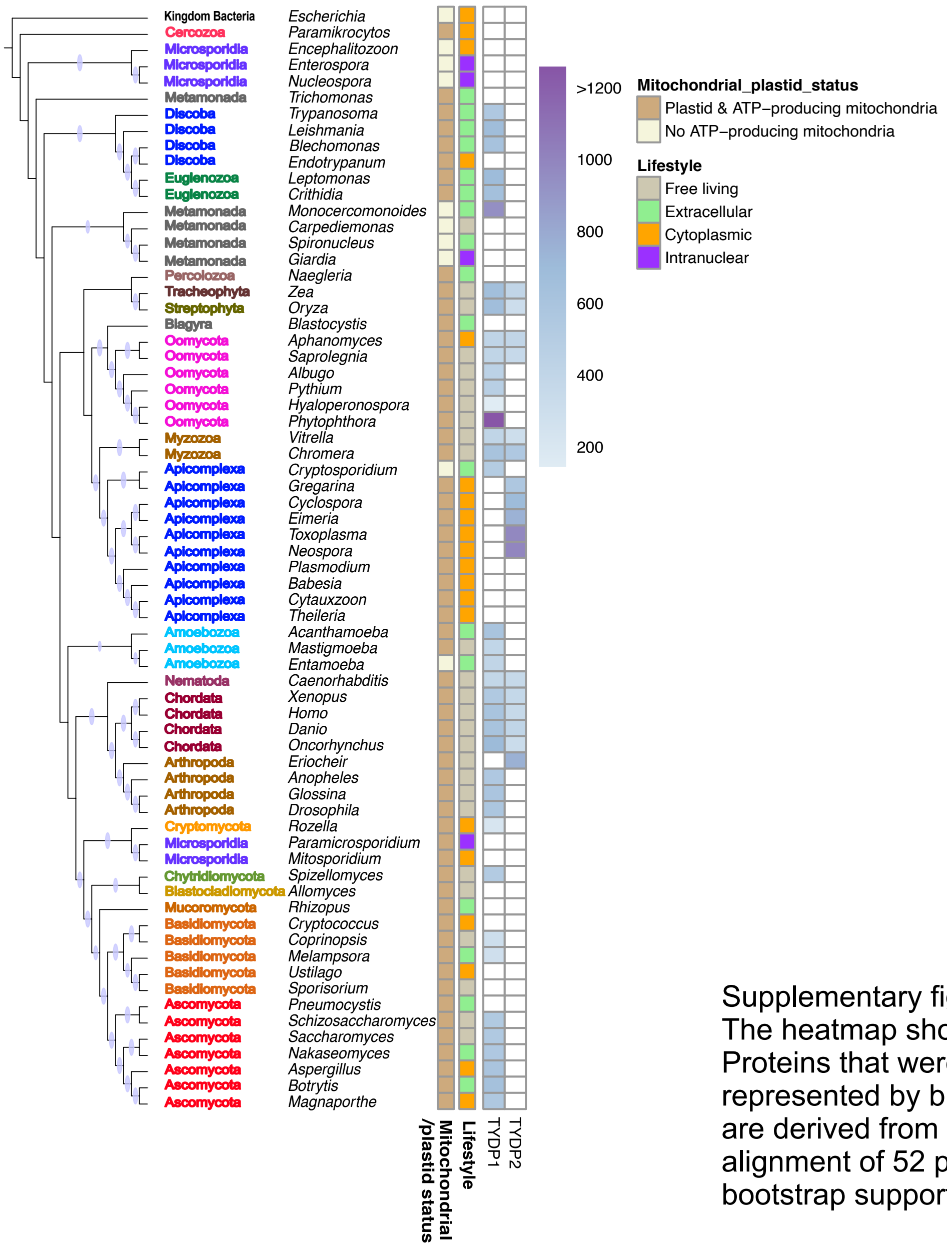

Supplementary fig 5: Profiling of topoisomerase damage reversal pathways. The heatmap shows the presence and length of constituent orthologs. Proteins that were not found to be encoded by the analysed genomes are represented by blank spaces. The phylogenetic positions in the cladogram are derived from maximum likelihood analyses performed on a concatenated alignment of 52 proteins. Oval symbols on phylogenetic tree represent bootstrap support values for the corresponding nodes greater than 70.

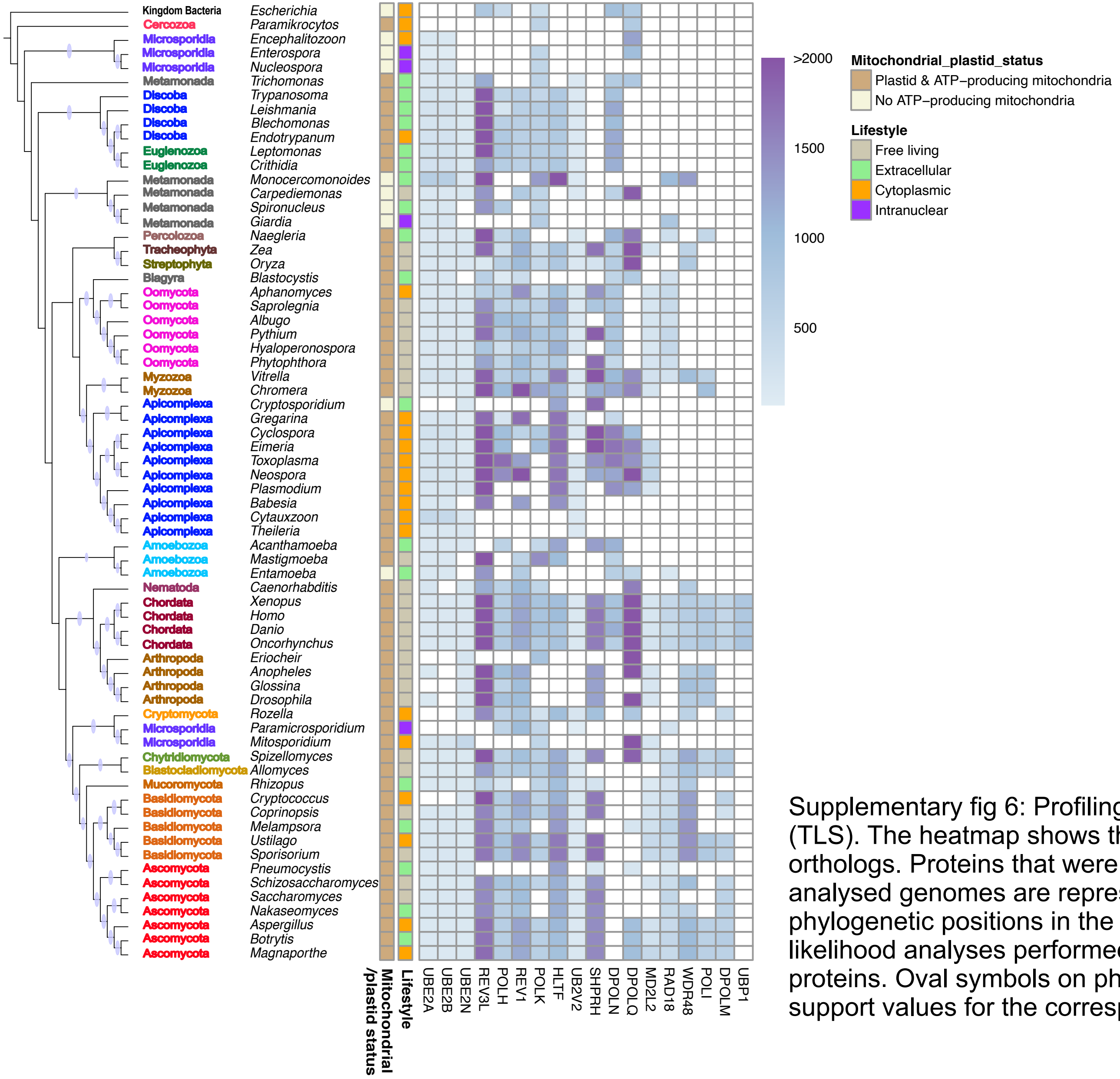

Supplementary fig 6: Profiling of Translesion synthesis pathways (TLS). The heatmap shows the presence and length of constituent orthologs. Proteins that were not found to be encoded by the analysed genomes are represented by blank spaces. The phylogenetic positions in the cladogram are derived from maximum likelihood analyses performed on a concatenated alignment of 52 proteins. Oval symbols on phylogenetic tree represent bootstrap support values for the corresponding nodes greater than 70.

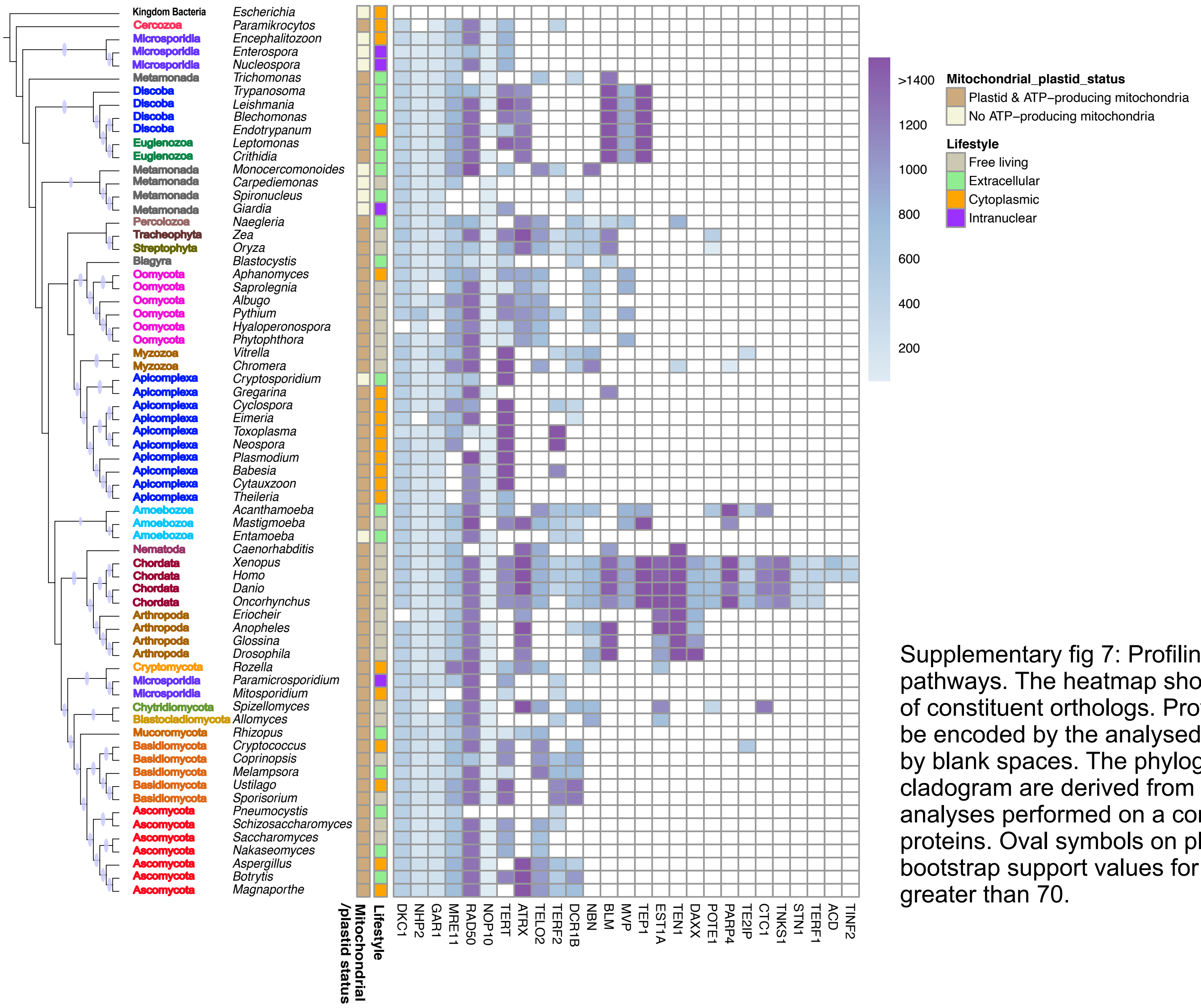

Supplementary fig 7: Profiling of Telomere maintenance pathways. The heatmap shows the presence and length of constituent orthologs. Proteins that were not found to be encoded by the analysed genomes are represented by blank spaces. The phylogenetic positions in the cladogram are derived from maximum likelihood analyses performed on a concatenated alignment of 52 proteins. Oval symbols on phylogenetic tree represent bootstrap support values for the corresponding nodes greater than 70.

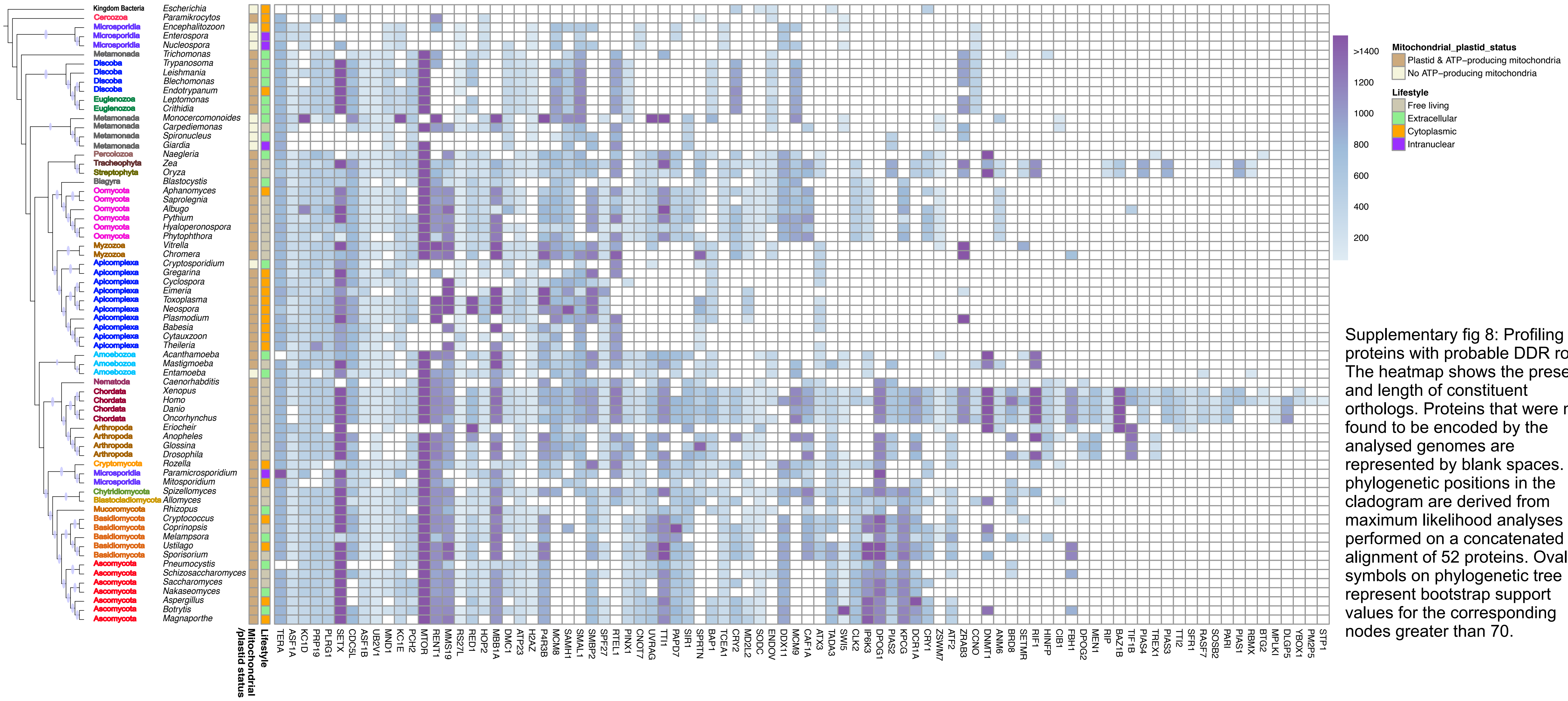

Supplementary fig 8: Profiling of proteins with probable DDR role. The heatmap shows the presence and length of constituent orthologs. Proteins that were not found to be encoded by the analysed genomes are represented by blank spaces. The phylogenetic positions in the cladogram are derived from maximum likelihood analyses performed on a concatenated alignment of 52 proteins. Oval symbols on phylogenetic tree represent bootstrap support values for the corresponding nodes greater than 70.

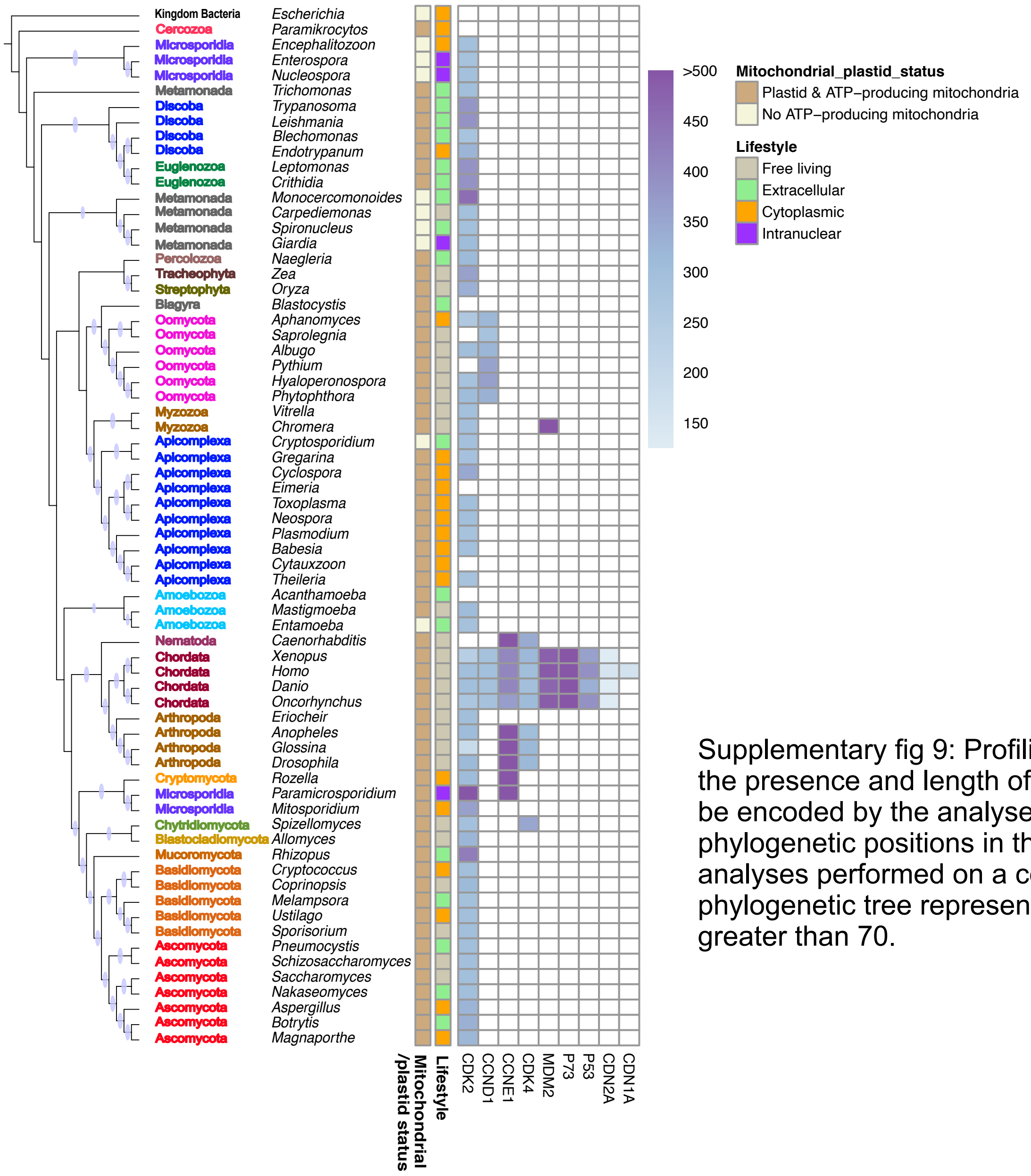

Supplementary fig 9: Profiling of proteins in the P53 pathways. The heatmap shows the presence and length of constituent orthologs. Proteins that were not found to be encoded by the analysed genomes are represented by blank spaces. The phylogenetic positions in the cladogram are derived from maximum likelihood analyses performed on a concatenated alignment of 52 proteins. Oval symbols on phylogenetic tree represent bootstrap support values for the corresponding nodes greater than 70.

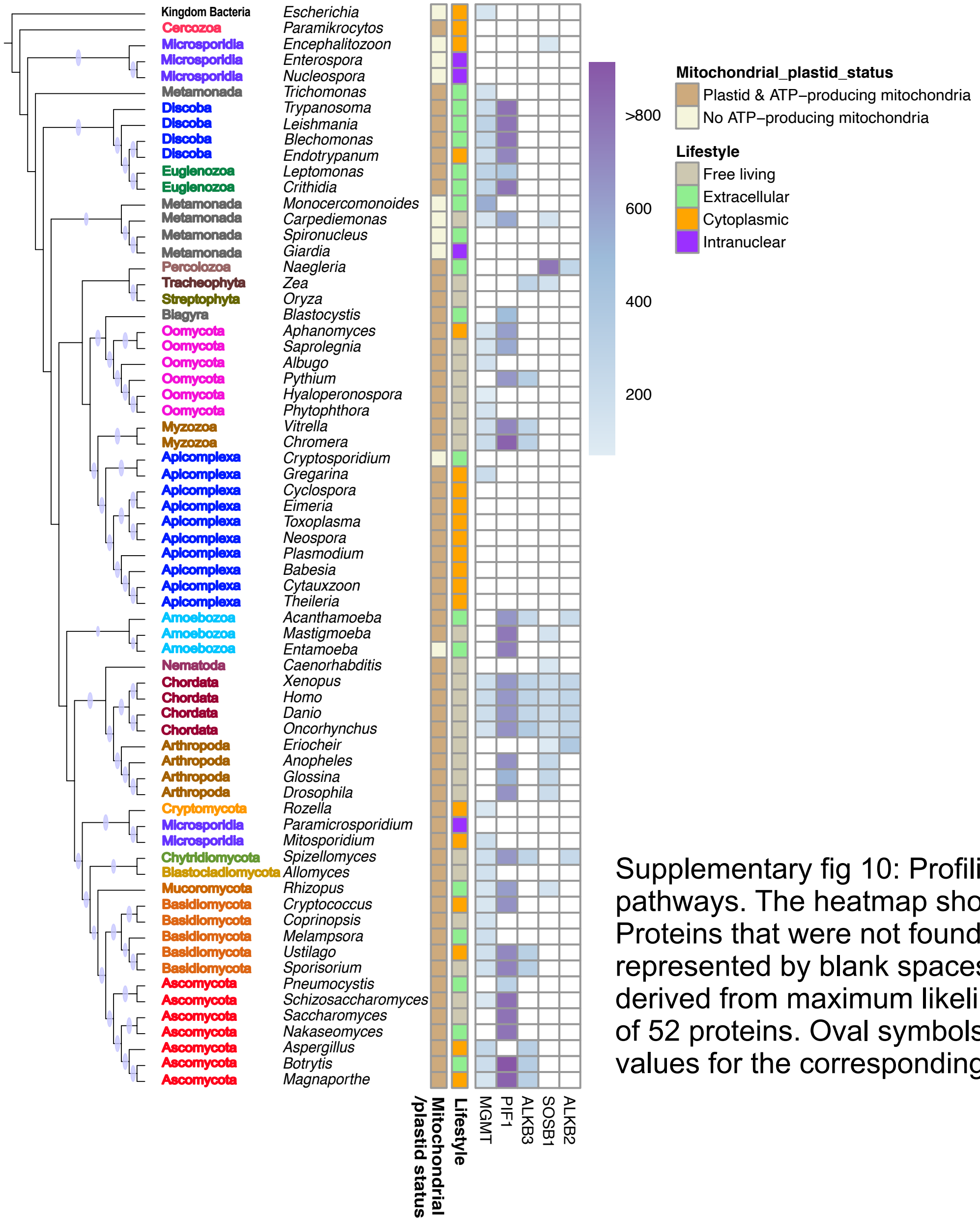

Supplementary fig 10: Profiling proteins involved in other single strand break repair pathways. The heatmap shows the presence and length of constituent orthologs. Proteins that were not found to be encoded by the analysed genomes are represented by blank spaces. The phylogenetic positions in the cladogram are derived from maximum likelihood analyses performed on a concatenated alignment of 52 proteins. Oval symbols on phylogenetic tree represent bootstrap support values for the corresponding nodes greater than 70.

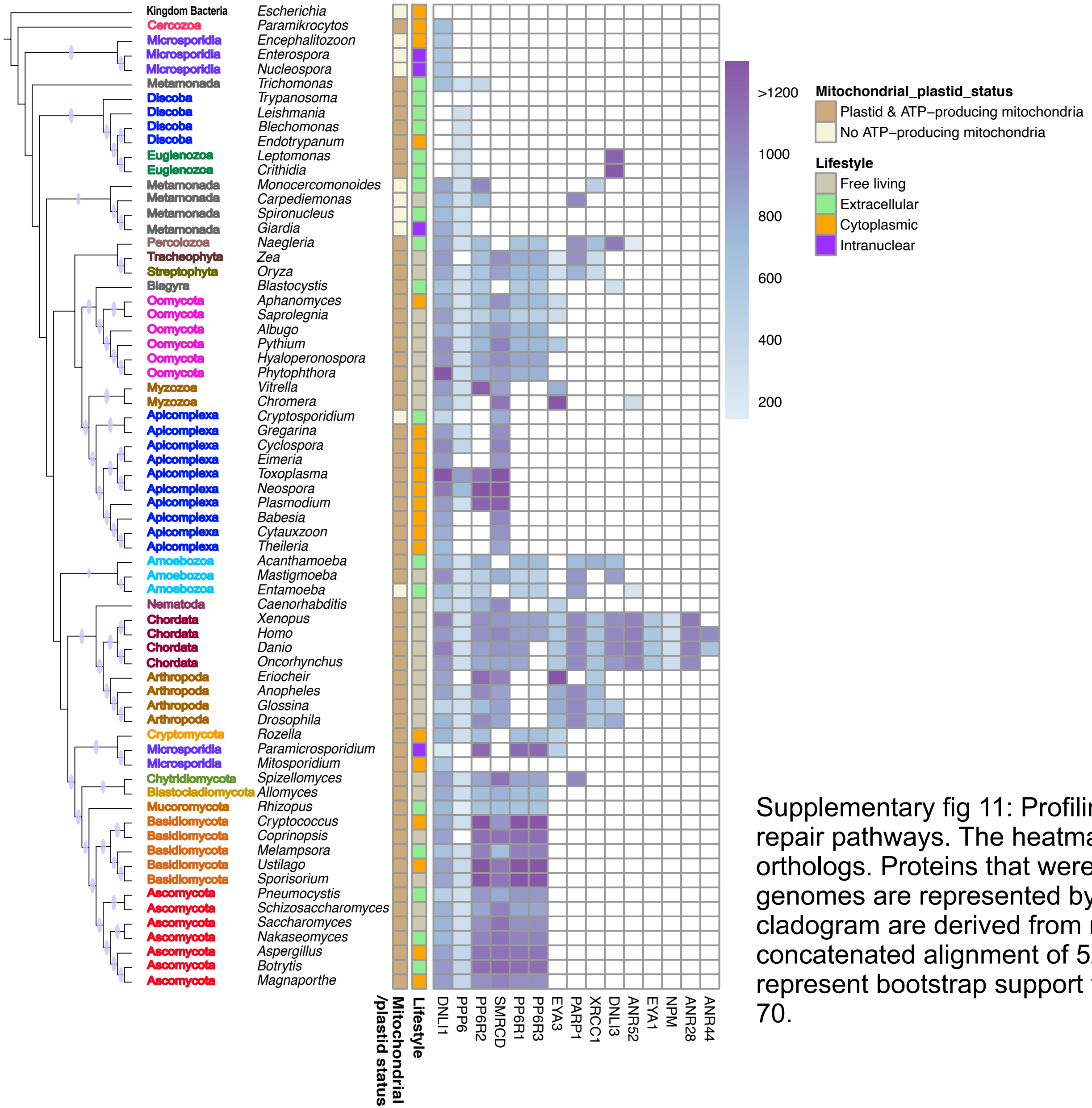

Supplementary fig 11: Profiling proteins involved in other double strand break repair pathways. The heatmap shows the presence and length of constituent orthologs. Proteins that were not found to be encoded by the analysed genomes are represented by blank spaces. The phylogenetic positions in the cladogram are derived from maximum likelihood analyses performed on a concatenated alignment of 52 proteins. Oval symbols on phylogenetic tree represent bootstrap support values for the corresponding nodes greater than 70.

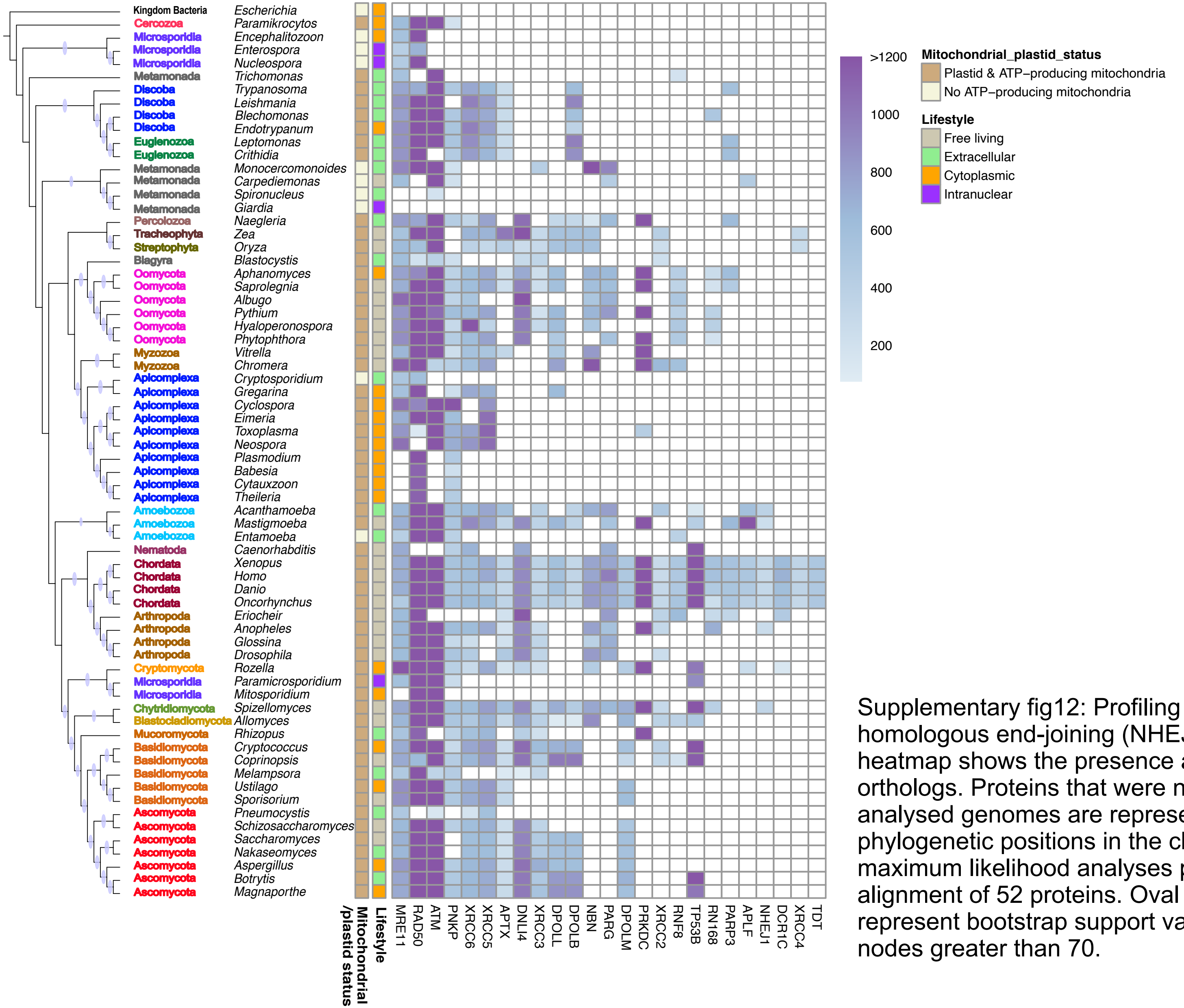

Supplementary fig12: Profiling proteins involved in non-homologous end-joining (NHEJ) repair pathways. The heatmap shows the presence and length of constituent orthologs. Proteins that were not found to be encoded by the analysed genomes are represented by blank spaces. The phylogenetic positions in the cladogram are derived from maximum likelihood analyses performed on a concatenated alignment of 52 proteins. Oval symbols on phylogenetic tree represent bootstrap support values for the corresponding nodes greater than 70.

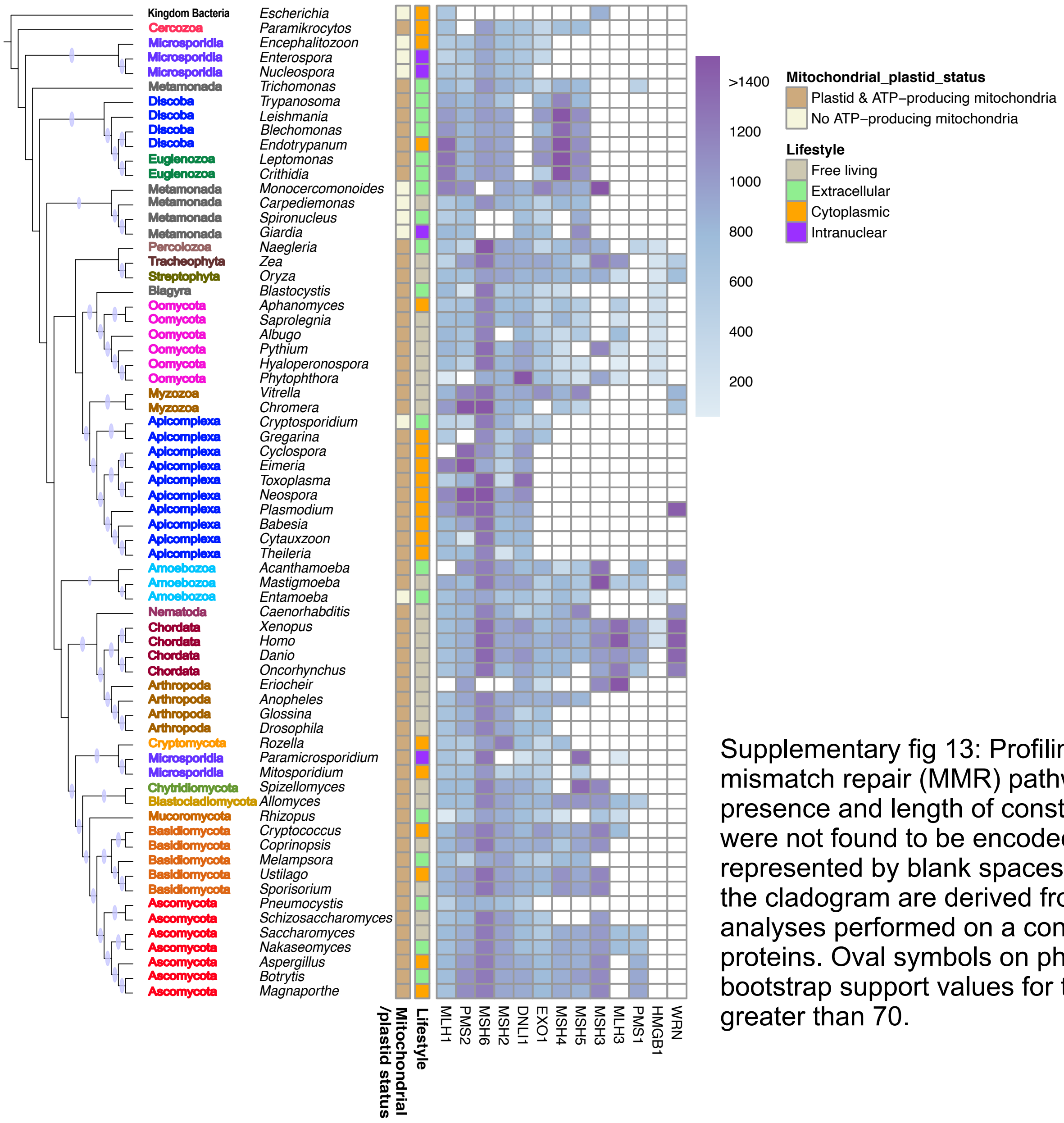

Supplementary fig 13: Profiling proteins involved in DNA mismatch repair (MMR) pathways. The heatmap shows the presence and length of constituent orthologs. Proteins that were not found to be encoded by the analysed genomes are represented by blank spaces. The phylogenetic positions in the cladogram are derived from maximum likelihood analyses performed on a concatenated alignment of 52 proteins. Oval symbols on phylogenetic tree represent bootstrap support values for the corresponding nodes greater than 70.

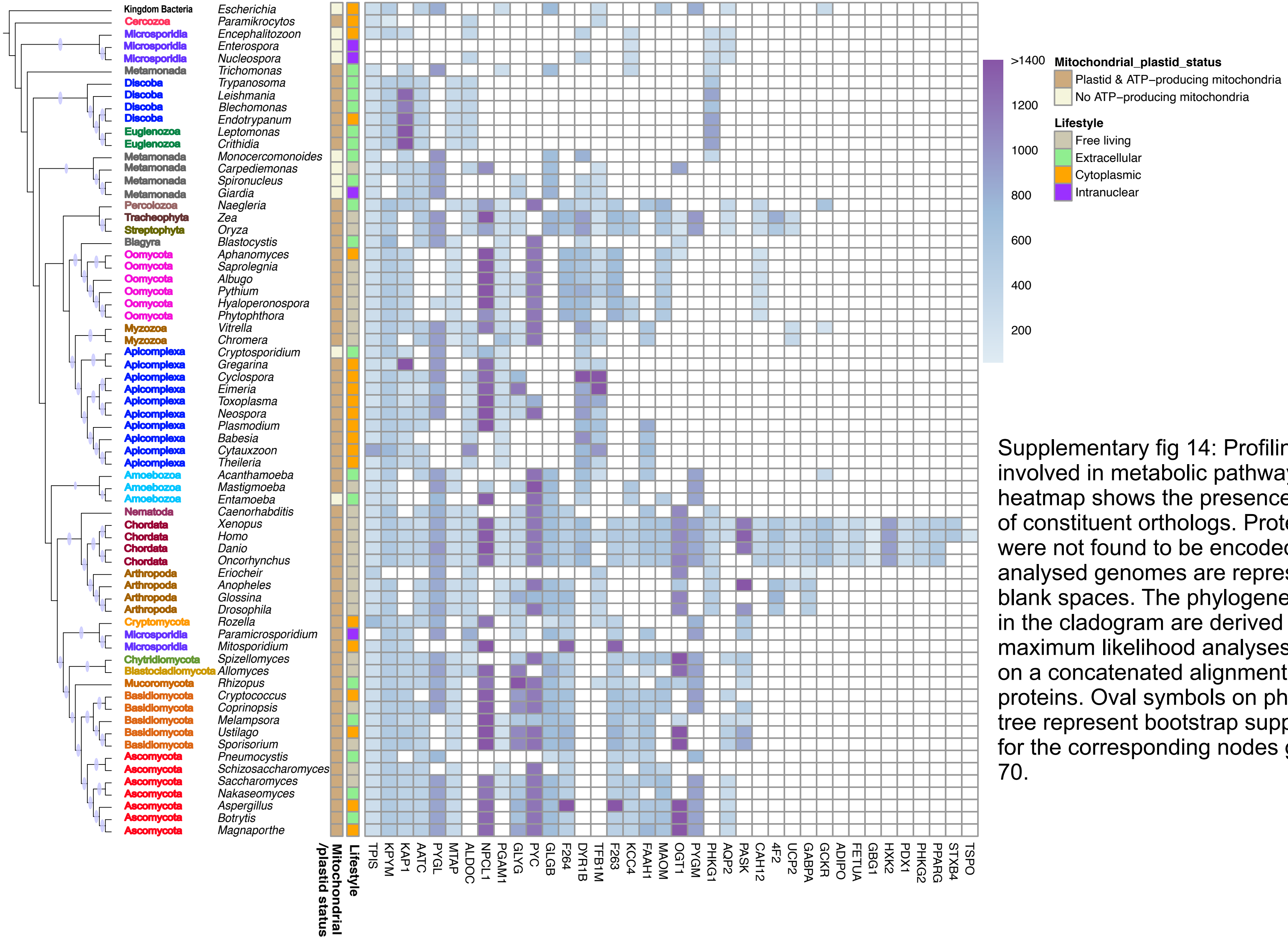

Supplementary fig 14: Profiling proteins involved in metabolic pathways. The heatmap shows the presence and length of constituent orthologs. Proteins that were not found to be encoded by the analysed genomes are represented by blank spaces. The phylogenetic positions in the cladogram are derived from maximum likelihood analyses performed on a concatenated alignment of 52 proteins. Oval symbols on phylogenetic tree represent bootstrap support values for the corresponding nodes greater than 70.

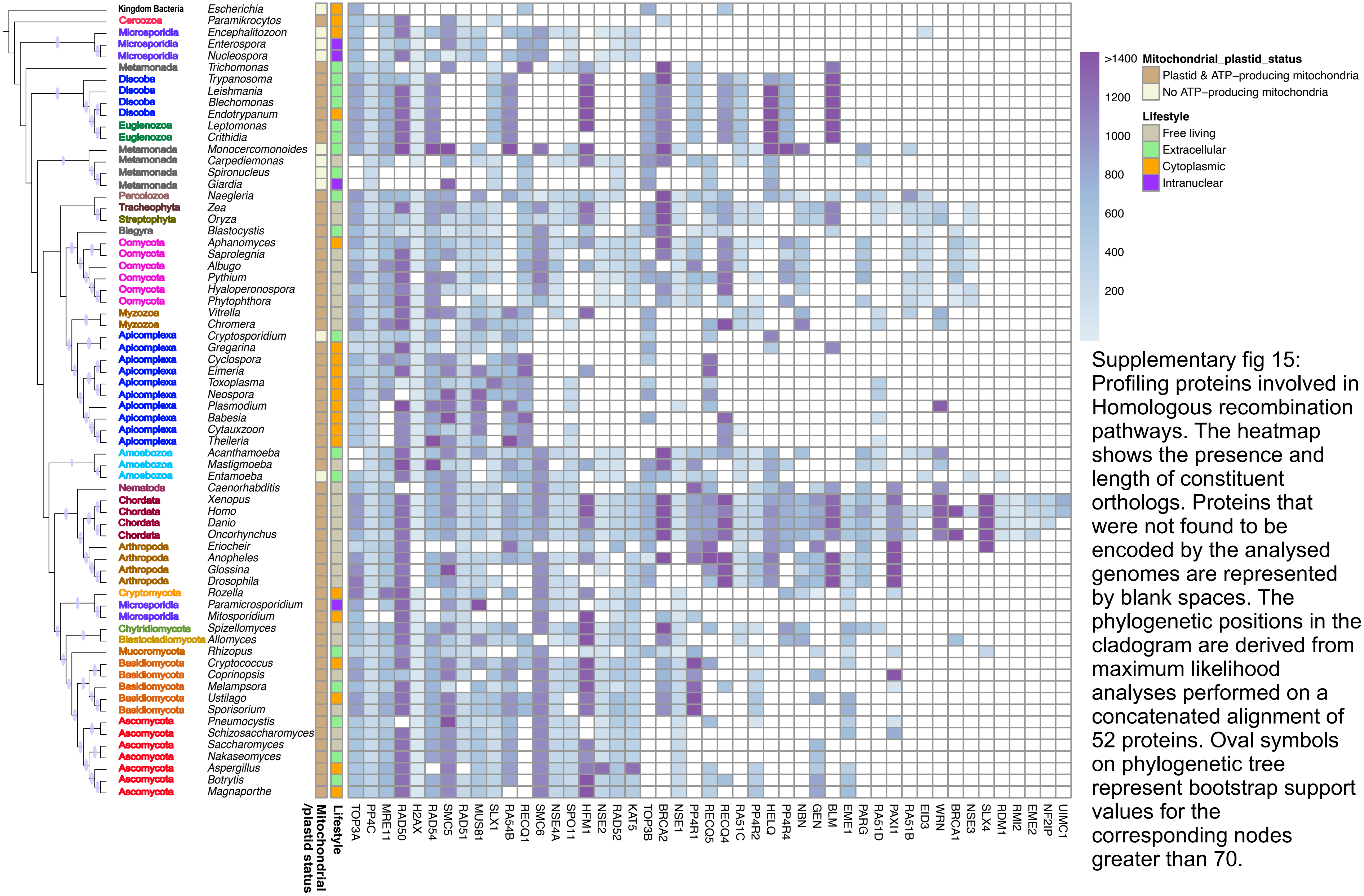

Supplementary fig 15: Profiling proteins involved in Homologous recombination pathways. The heatmap shows the presence and length of constituent orthologs. Proteins that were not found to be encoded by the analysed genomes are represented by blank spaces. The phylogenetic positions in the cladogram are derived from maximum likelihood analyses performed on a concatenated alignment of 52 proteins. Oval symbols on phylogenetic tree represent bootstrap support values for the corresponding nodes greater than 70.

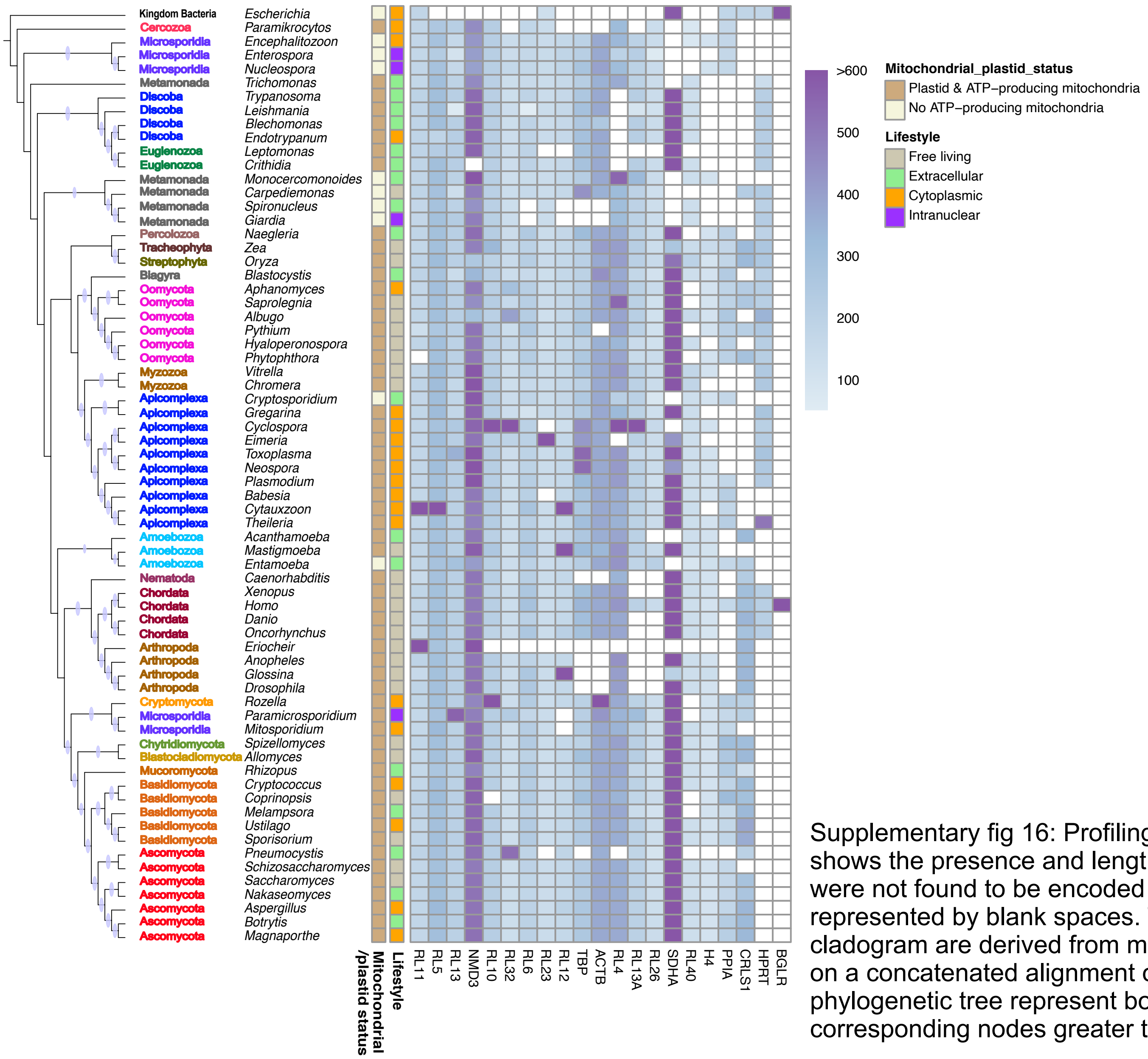

Supplementary fig 16: Profiling housekeeping proteins. The heatmap shows the presence and length of constituent orthologs. Proteins that were not found to be encoded by the analysed genomes are represented by blank spaces. The phylogenetic positions in the cladogram are derived from maximum likelihood analyses performed on a concatenated alignment of 52 proteins. Oval symbols on phylogenetic tree represent bootstrap support values for the corresponding nodes greater than 70.

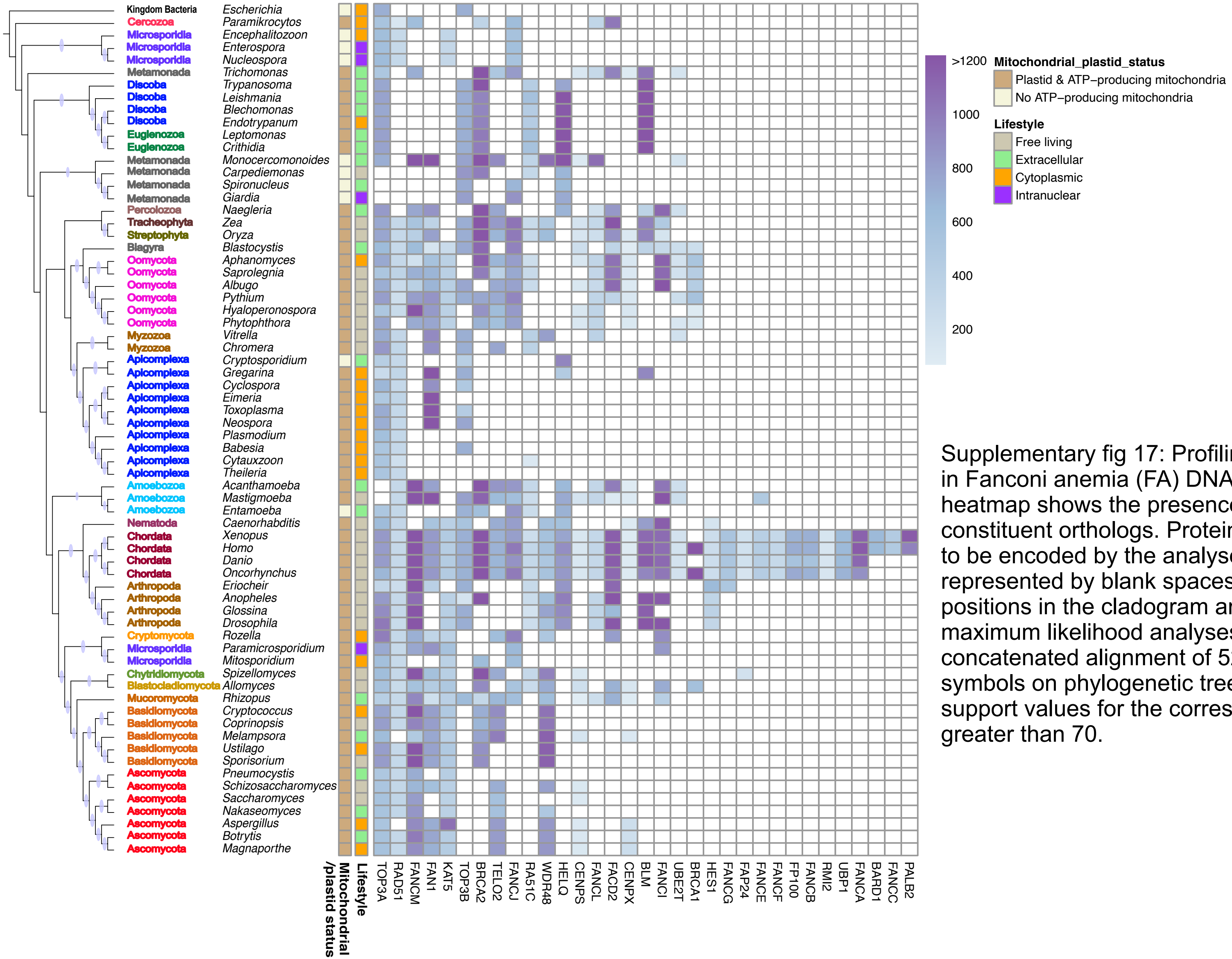

Supplementary fig 17: Profiling proteins involved in Fanconi anemia (FA) DNA repair pathways. The heatmap shows the presence and length of constituent orthologs. Proteins that were not found to be encoded by the analysed genomes are represented by blank spaces. The phylogenetic positions in the cladogram are derived from maximum likelihood analyses performed on a concatenated alignment of 52 proteins. Oval symbols on phylogenetic tree represent bootstrap support values for the corresponding nodes greater than 70.

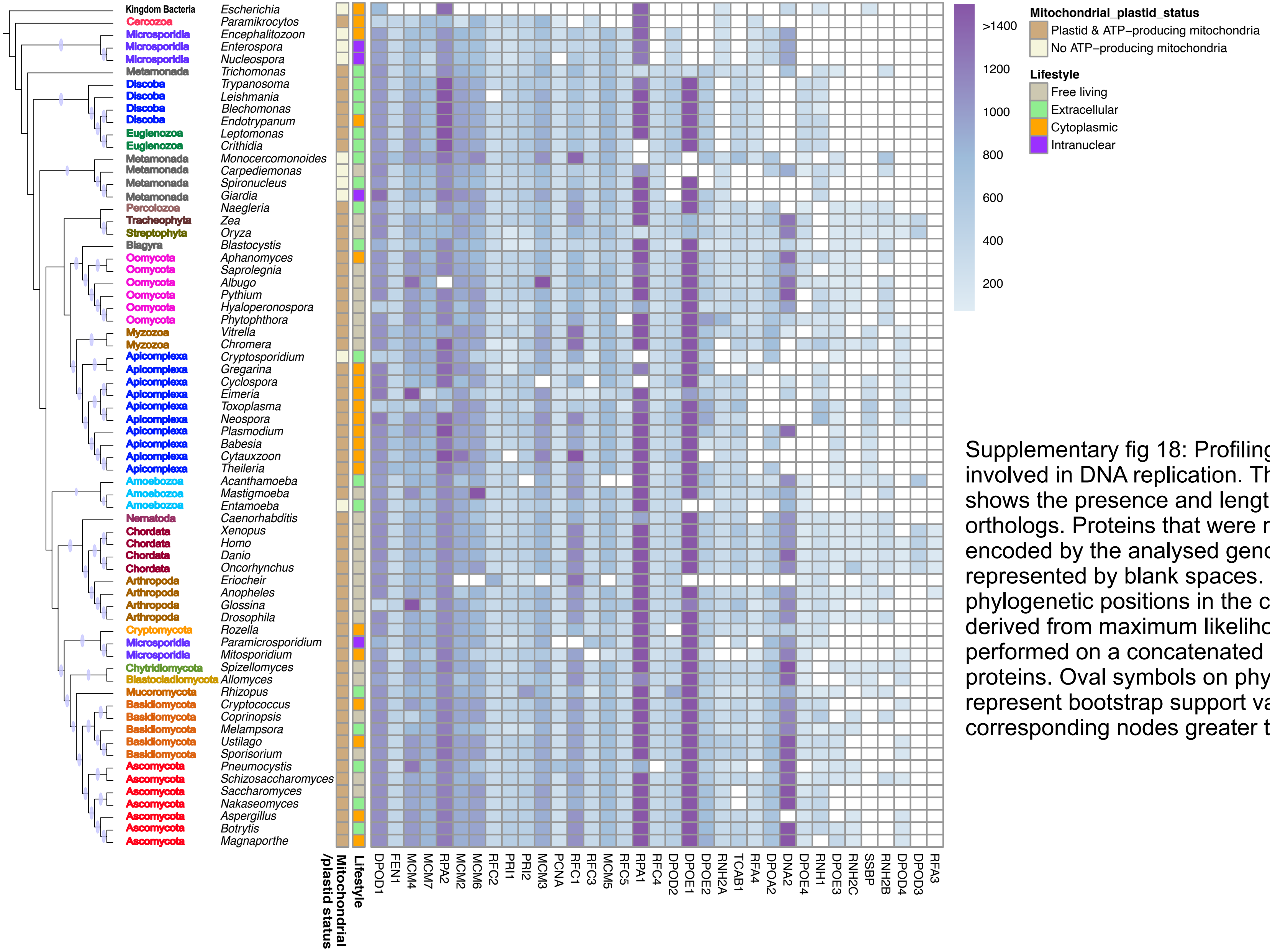

Supplementary fig 18: Profiling proteins involved in DNA replication. The heatmap shows the presence and length of constituent orthologs. Proteins that were not found to be encoded by the analysed genomes are represented by blank spaces. The phylogenetic positions in the cladogram are derived from maximum likelihood analyses performed on a concatenated alignment of 52 proteins. Oval symbols on phylogenetic tree represent bootstrap support values for the corresponding nodes greater than 70.

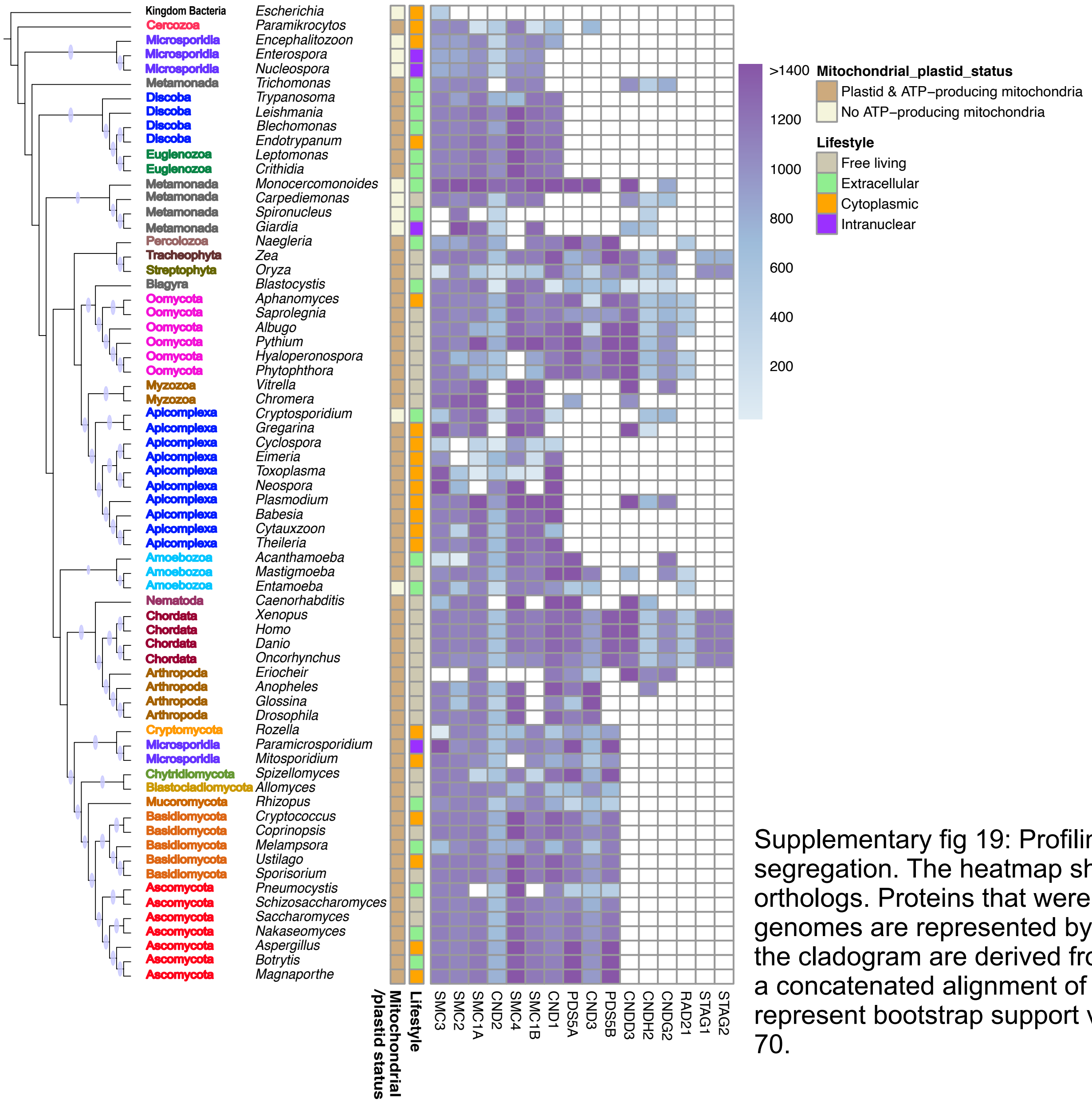

Supplementary fig 19: Profiling proteins involved in chromosome segregation. The heatmap shows the presence and length of constituent orthologs. Proteins that were not found to be encoded by the analysed genomes are represented by blank spaces. The phylogenetic positions in the cladogram are derived from maximum likelihood analyses performed on a concatenated alignment of 52 proteins. Oval symbols on phylogenetic tree represent bootstrap support values for the corresponding nodes greater than 70.

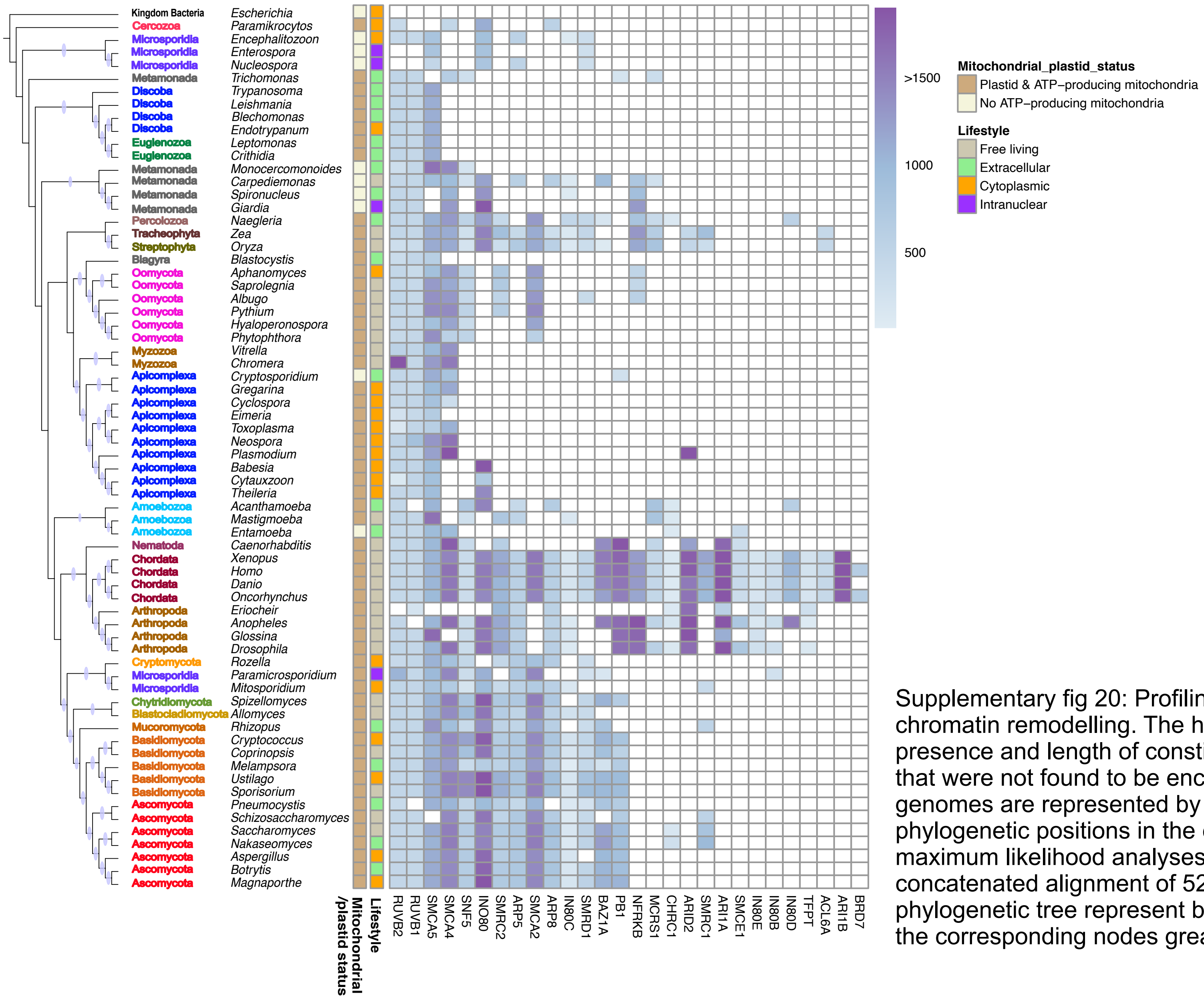

Supplementary fig 20: Profiling proteins involved in chromatin remodelling. The heatmap shows the presence and length of constituent orthologs. Proteins that were not found to be encoded by the analysed genomes are represented by blank spaces. The phylogenetic positions in the cladogram are derived from maximum likelihood analyses performed on a concatenated alignment of 52 proteins. Oval symbols on phylogenetic tree represent bootstrap support values for the corresponding nodes greater than 70.

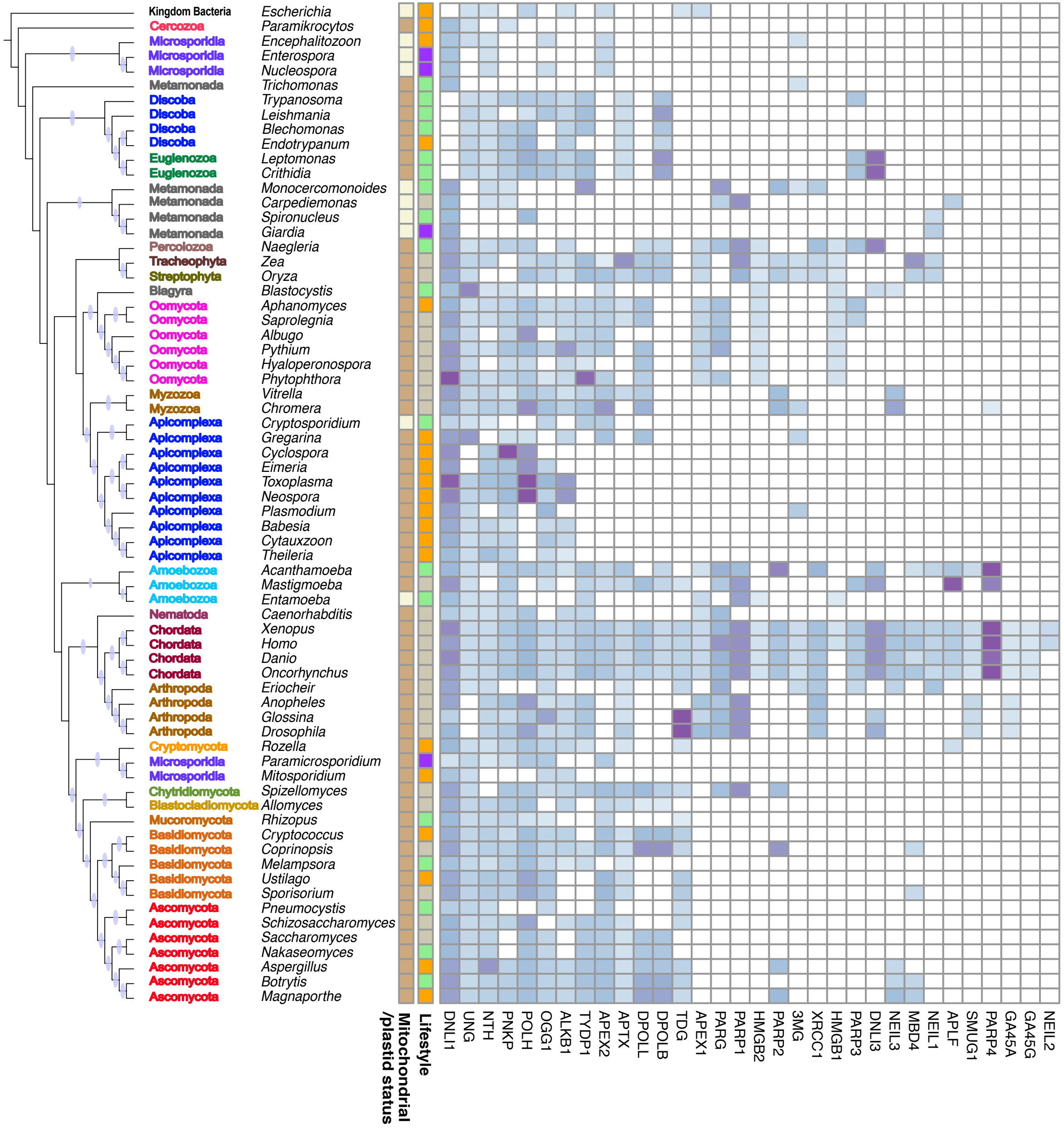

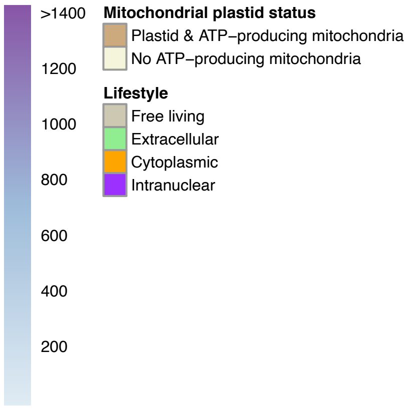

Supplementary fig 21: Profiling proteins involved in base excision repair. The heatmap shows the presence and length of constituent orthologs. Proteins that were not found to be encoded by the analysed genomes are represented by blank spaces. The phylogenetic positions in the cladogram are derived from maximum likelihood analyses performed on a concatenated alignment of 52 proteins. Oval symbols on phylogenetic tree represent bootstrap support values for the corresponding nodes greater than 70.

Supplementary figure 22: Comparing the protein length between microsporidia that possess ATP-producing mitochondria (Paramicrosporidia) and microsporidia that lack ATP producing mitochondria (Encephalitozoon, Enterospora and Nucleospora)

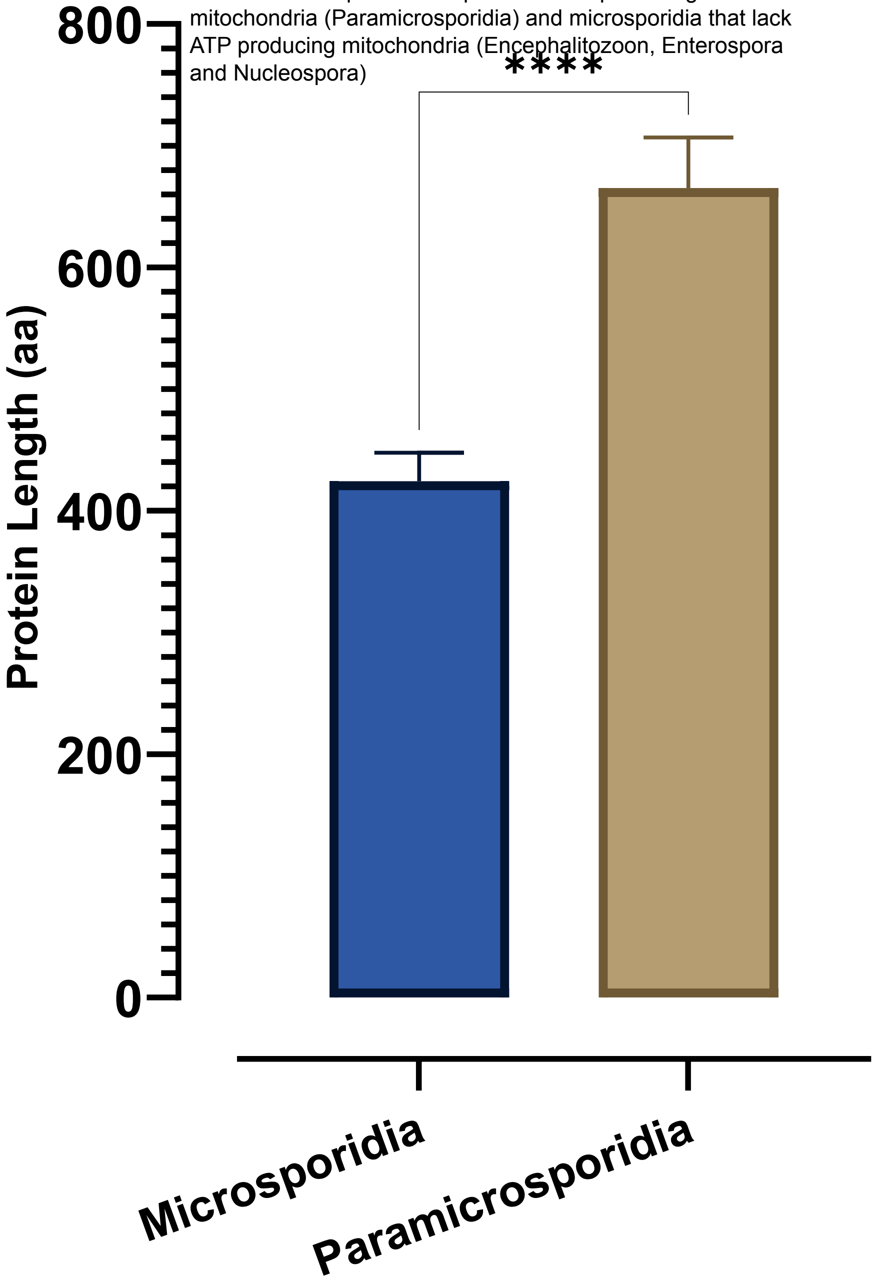

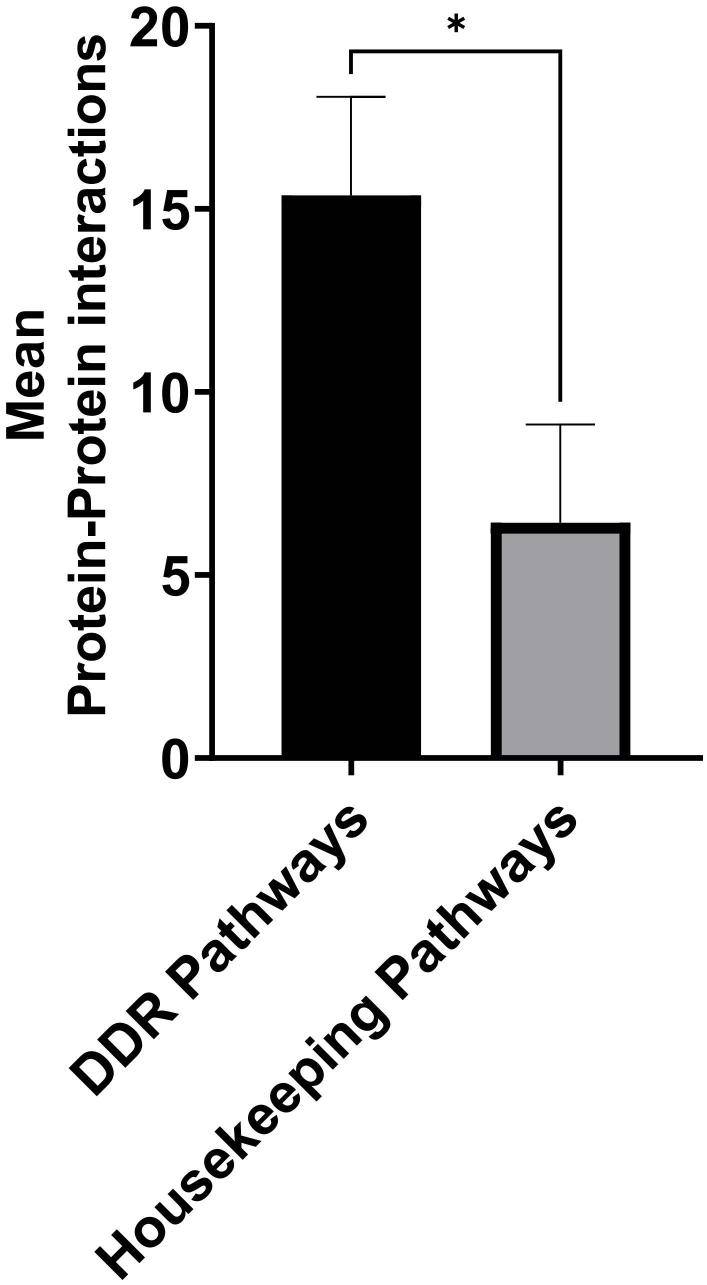

Supplementary figure 23: Protein-Protein Interactions predicted by the STRING database for 54 DDR and Housekeeping proteins showing that DDR proteins to have more protein-protein interactions than housekeeping proteins

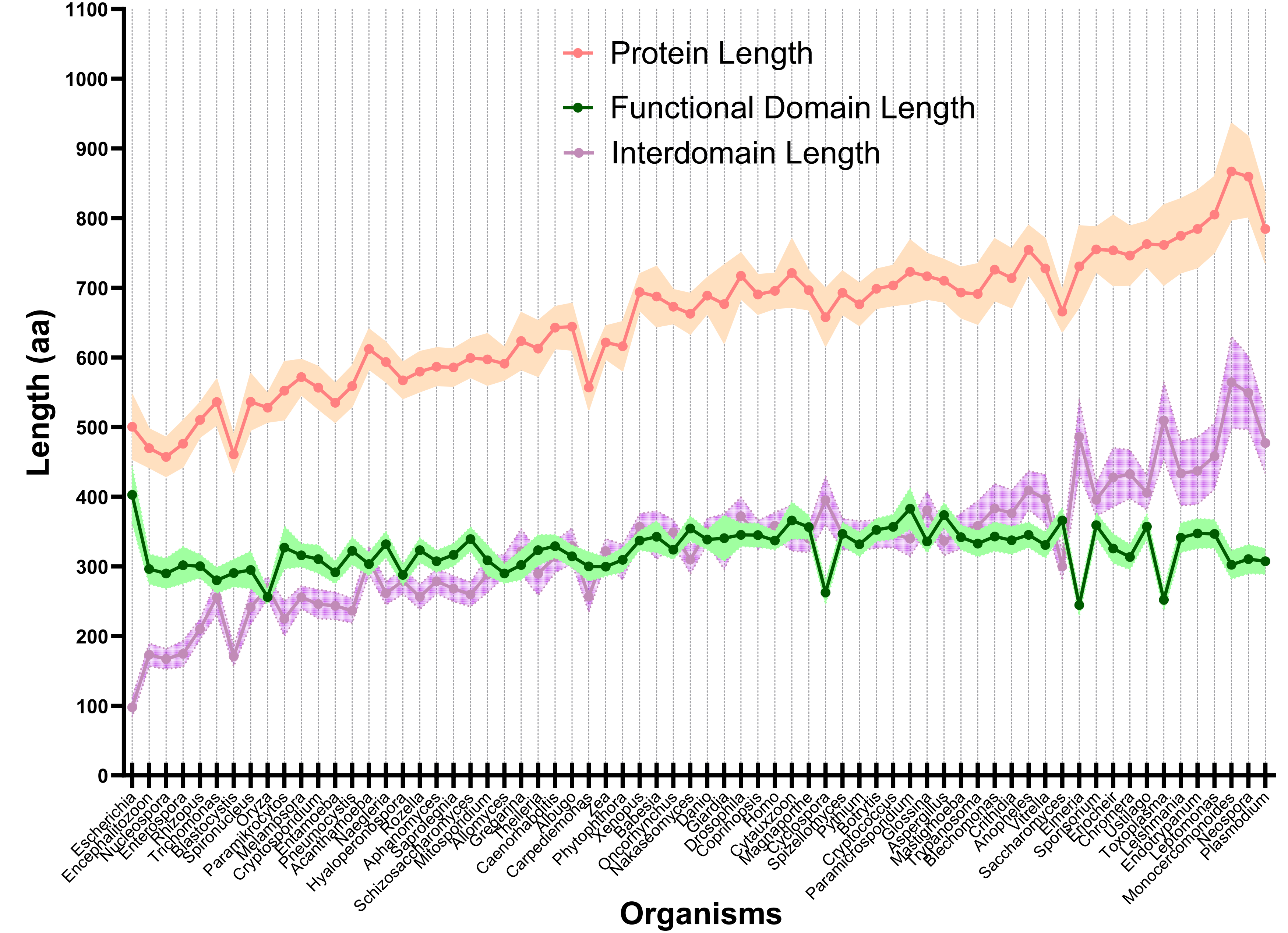

Supplementary figure 24: Mapping protein length to functional domain and interdomain lengths of 526 proteins shows that there is a strong positive correlation between interdomain length and protein length.

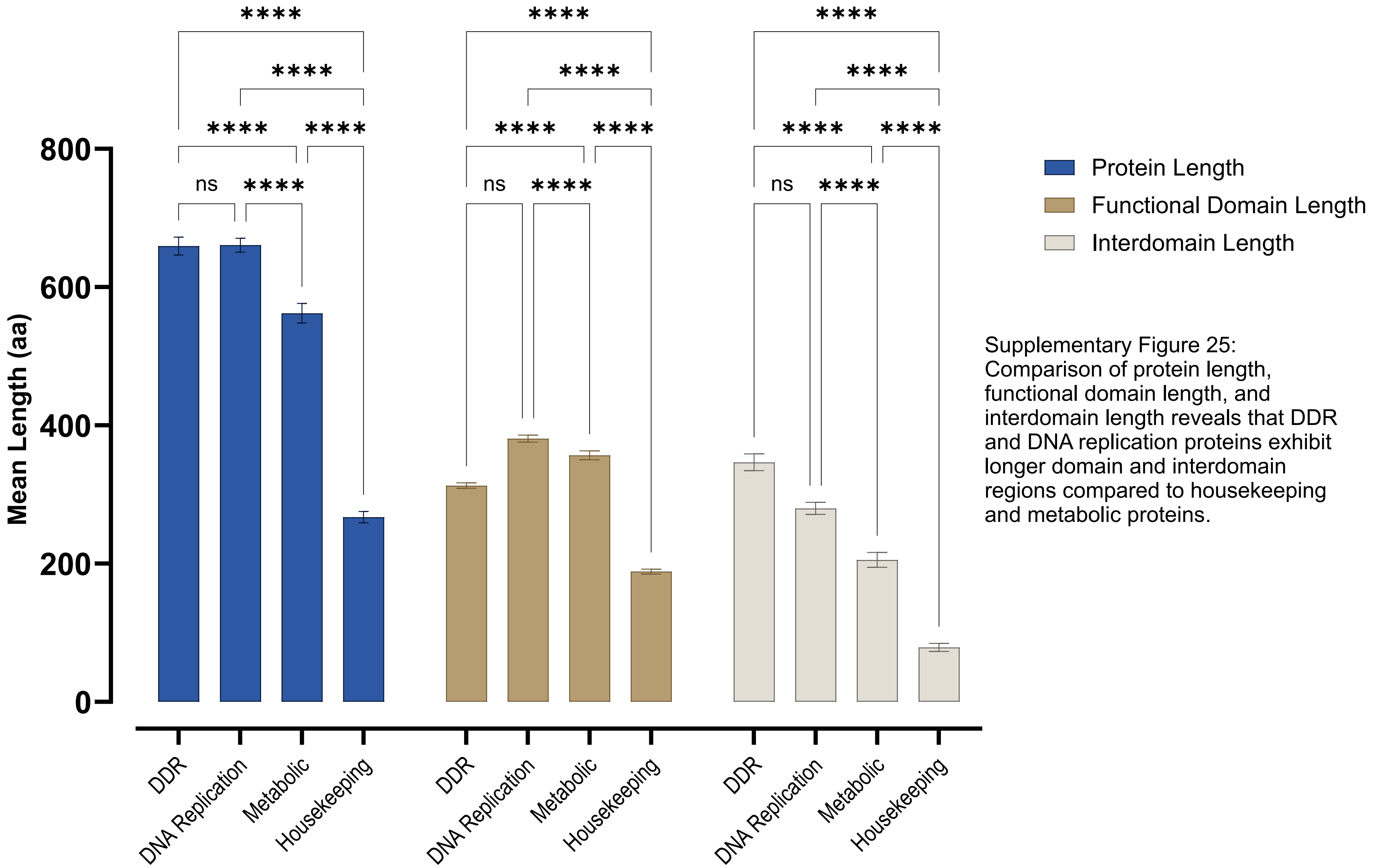

Supplementary Figure 25: Comparison of protein length, functional domain length, and interdomain length reveals that DDR and DNA replication proteins exhibit longer domain and interdomain regions compared to housekeeping and metabolic proteins.

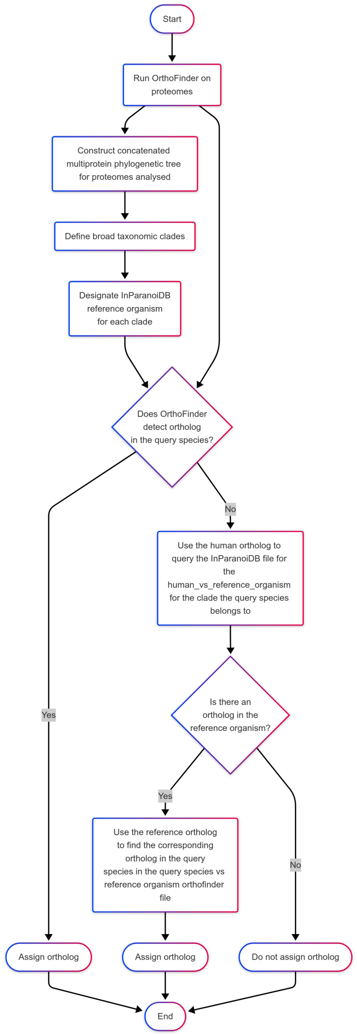

Supplementary Figure 26: Flow diagram showing ortholog clustering methodology
