# Supplementary material for: Evolutionary Insights into the Length Variation of DNA Damage Response Proteins Across Eukaryotes
Source: Genome Biol Evol. 2025 May 19;17(6):evaf089. doi: 10.1093/gbe/evaf089 (PMC12134460; doi:10.1093/gbe/evaf089)
Supplement: evaf089_Supplementary_Data [file evaf089_supplementary_data.zip › Supplementary table captions.docx]

Supplementary table 1: Cross-referencing the largest protein within the most conserved ortholog set for each pathway.

Supplementary table 2: Preservation of the various pathways investigated in this manuscript

Supplementary table 3: list of type III RNRs found in *Aphanomyces*, *Acanthamoeba*, *Phytophthora*, *Naegleria*, *Allomyces*, *Blastocystis, Monocercomonoides, Pythium* and *Saprolegnia.*

Supplementary table 4: List of organisms whose proteomes were used in this study

Supplementary table 5: Protein structure predictions of a selection of unusually long proteins
